# How often do general practitioners use placebos and non-specific interventions? Systematic review and meta-analysis of surveys

**DOI:** 10.1371/journal.pone.0202211

**Published:** 2018-08-24

**Authors:** Klaus Linde, Oxana Atmann, Karin Meissner, Antonius Schneider, Ramona Meister, Levente Kriston, Christoph Werner

**Affiliations:** 1 Technical University of Munich, TUM School of Medicine, Institute of General Practice, Munich, Germany; 2 Division of Health Promotion, University of Applied Sciences Coburg, Coburg, Germany; 3 Institute of Medical Psychology, Faculty of Medicine, LMU Munich, Munich, Germany; 4 Department of Medical Psychology, University Medical Center Hamburg-Eppendorf, Hamburg, Germany; Robert Gordon University, UNITED KINGDOM

## Abstract

**Background:**

In a systematic review and meta-analysis we summarize the available evidence on how frequently general practitioners/family physicians (GPs) use pure placebos (e.g., placebo pills) and non-specific therapies (sometimes referred to as impure placebos; e.g., antibiotics for common cold).

**Methods:**

We searched Medline, PubMed and SCOPUS up to July 2018 to identify cross-sectional quantitative surveys among GPs. Outcomes of primary interest were the percentages of GPs having used any placebo, pure placebos or non-specific therapies at least once in their career, at least once in the last year, at least monthly or at least weekly. Outcomes were described as proportions and pooled with random-effects meta-analysis.

**Results:**

Of 674 publications, 16 studies from 13 countries with a total of 2.981 participating GPs (range 27 to 783) met the inclusion criteria. The percentage of GPs having used any form of placebo at least once in their career ranged from 29% to 97%, in the last year at least once from 46% to 95%, at least monthly from 15% to 89%, and at least weekly from 1% to 75%. The use of non-specific therapies by far outnumbered the use of pure placebo. For example, the proportion of GPs using pure placebos at least monthly varied between 2% and 15% compared to 53% and 89% for non-specific therapies; use at least weekly varied between 1% and 3% for pure placebos and between 16% and 75% for non-specific therapies. Besides eliciting placebos effects, many other reasons related to patient expectations, demands and medical problems were reported as reasons for applying placebo interventions.

**Conclusion:**

High prevalence estimates of placebo use among GPs are mainly driven by the frequent use of non-specific therapies; pure placebos are used rarely. The interpretation of our quantitative findings is complicated by the diversity of definitions and survey methods.

## Introduction

Although the use of placebo interventions outside clinical trials without full informed consent is generally considered unethical [1–3], surveys in various countries show that many physicians prescribe “placebos” in routine clinical practice [4–7]. A major problem when investigating the prevalence of placebo use is that it is far from clear what kind of intervention qualifies as a placebo in routine clinical practice [8,9]. There is little disagreement about what is called “pure placebo”, such as sugar pills or saline injections. However, placebo definitions in most surveys also include many potentially active interventions, which are thought to have no specific activity on the condition being treated beyond a placebo effect [4–6]. These interventions sometimes are called “impure placebos” [4,6] or “non-specific therapies” [7]. To emphasize the clinically important difference to “pure placebos” we prefer the term “non-specific therapies” in this report. Typical examples are antibiotics in viral infections or vitamins in patients without deficiency, but some surveys also included treatments not backed up by solid evidence, positive suggestions or non-essential physical or technical examinations.

In recent years, the use of placebos has been investigated more often among general practitioners (GPs) than among any other medical disciplines [4–7,10]. It seems plausible to assume that GPs use placebos more frequently because they see many patients with unclear, non-specific complaints or minor ailments as well as chronically ill patients coming back from specialists without a fully satisfying therapy. In this report, we present a systematic review and meta-analysis of all available cross-sectional quantitative surveys on the use of placebo interventions among general practitioners. Our objective was to summarize current knowledge on 1) the frequency of use of any placebo interventions, pure placebos and non-specific therapies; 2) what kind of placebo interventions actually are used; and 3) whether placebo use among GPs differs from that in other medical specialties.

## Methods

The methods of the review were pre-defined in a protocol (see S1 File); we have considered registering our protocol in PROSPERO, but this register is limited to reviews with “a health related outcome” (https://www.crd.york.ac.uk/prospero/aboutreg.php?reg=inclusioncriteria).

### Selection criteria, literature search and selection process

To be included, studies had to be cross-sectional quantitative surveys among GPs (defined as physicians explicitly described as GPs or family physicians, or in countries without such a specialization primary care physicians seeing unselected adult patients or patients of any age). Surveys in mixed samples of physicians were included if separate subgroup data on GPs was reported in the publication or could be obtained from authors or from re-analyses of the raw data. For inclusion in our review, publications also had to report numerical results on the use of any placebo intervention, or pure placebos, or non-specific therapies. We used the following definitions for our key terms: placebo interventions—any intervention considered a placebo (pure or impure) in a primary study. Pure placebos—any products such as placebo tablets or pure placebo pills without active agent and manufactured to be a placebo intervention; saline injections or infusions provided as placebos. Non-specific therapies (= impure placebo)—“placebo” interventions other than pure placebos, e.g. antibiotics in viral infections not considered indicated by the provider.

The main electronic literature searches were performed in February 2017 in PubMed, Medline (using Web of Science) and Scopus (see S2 File for detailed strategies). PubMed was searched using a strategy combining a subject term (“placebo” in title), a combination of design terms (“survey” and related terms) and a combination of field terms (“general practice” and related terms). As this strategy failed to identify two relevant surveys known to the authors, an additional Medline search focusing mainly on title words (excluding publications likely to be placebo-controlled trials) was performed. Scopus was searched using a similar strategy. In addition, citation searches were done in Google Scholar for four key publications [4,10–12]. The final update search was performed in July 2018. Based on a previous comprehensive systematic review of the use of placebos or placebo effects in clinical practice in general [4], involving three of the authors (KL, KM, AS), we knew that no potentially eligible studies were published before 1997. Therefore, we limited our literature search to publications published after 1996.

At least two reviewers independently screened search hits (titles and abstracts) from electronic databases for potentially eligible publications. References identified in the Google Scholar citation searches were screened by a single reviewer. Clearly irrelevant search hits were excluded. Publications considered potentially eligible by at least one reviewer were obtained in full text. All potentially relevant full text publications were checked formally against the selection criteria by at least two reviewers. Disagreements were resolved by discussion.

### Data collection and quality assessment

Information on country of the survey, population, sampling, definitions, methods and findings was extracted using a pretested form by at least two reviewers independently. Following a screening of how data was reported in publications, we extracted the following data to estimate the frequency of placebo use: number of participating physicians analyzed; number of physicians having used any type of placebo intervention, pure placebos, and non-specific specific therapies (three intervention classes) at least once in their career, in the last 12 months at least once, at least monthly, and at least weekly (four timeframes). This resulted in a total of 12 outcomes (three intervention classes X four different timeframes). In addition we extracted the number of physicians having used defined placebo interventions (e.g., placebo pills and similar products, sodium chloride (NaCl) injections or infusions for placebo purposes, antibiotics for viral infections) in their career. If data for other physician groups beside GPs were reported, these data were extracted too. If included publications did not report the outcomes listed above although they were measured or probably measured, we tried to obtain these from the authors. In addition, one reviewer extracted and classified the reasons for giving placebos as reported in the included studies.

We assessed quality using six criteria adapted from a widely used tool for observational studies [13] and the quality assessment tool used in a recent major meta-analysis of cross-sectional surveys [14]: 1) Was the underlying population adequately defined and relevant? 2) Was the procedure to draw a sample from the population adequate? 3) Is the response rate sufficiently high to rule out selection bias? 4) Did more than 200 GPs participate? 5) Was there some systematic pre-testing or validation of the questionnaire? 6) Were participating GPs described? Scoring was operationalized by detailed instructions (see S1 File). We considered the questions 1, 2 and 3 as key questions of methodological quality. We further summarized the answers to sampling (questions 1 and 2) and considered studies with a random sampling of GPs from an adequately defined (e.g., a country of a region) and sufficiently large population (more than 500 GPs) being of high quality. We considered response rates (number of participating GPs divided by the number of GP invited) above 0.7 high quality, between 0.4 and 0.7 as moderate quality and below 0.4 as low quality. Disagreements in the extraction and assessment process were resolved by discussion.

### Data analysis

Outcome data on placebo use were extracted as absolute frequencies (number of physicians meeting a defined criterion). For obtaining proportions, these numbers were divided by the number of participating physicians. Missing answers were counted as not meeting the criterion (in studies reporting the number of missing observations these were always below 10%). Proportions were transformed into logits (logarithmic odds) and the respective standard errors and 95% confidence intervals were calculated for all studies and outcomes. The logits were used for meta-analyses and back transformed into proportions and percentages afterwards. Based on the limited available data, we only compared the use of any placebo at any time in the career between GPs, specialists in private practice, hospital physicians, pediatricians, and internists providing primary care. All comparisons were direct (comparison of GPs and the other discipline in the same survey). In each study comparing GPs to other groups, odds ratios with 95% confidence intervals were calculated from the two proportions. The analyses were performed on the log-scale and the meta-analytic results were back transformed to the odds ratio scale for interpretation. All analyses were random-effects meta-analyses performed using the restricted maximum likelihood estimator. The extent of statistical heterogeneity (variation of study findings beyond chance) was tested for significance using Cochrane´s Q-test and quantified by means of the I^2^ statistic. All analyses were performed in the open source statistical environment R with the packages metaphor and ggplot2 [15].

## Results

### Literature search and selection process

**Fig 1:**
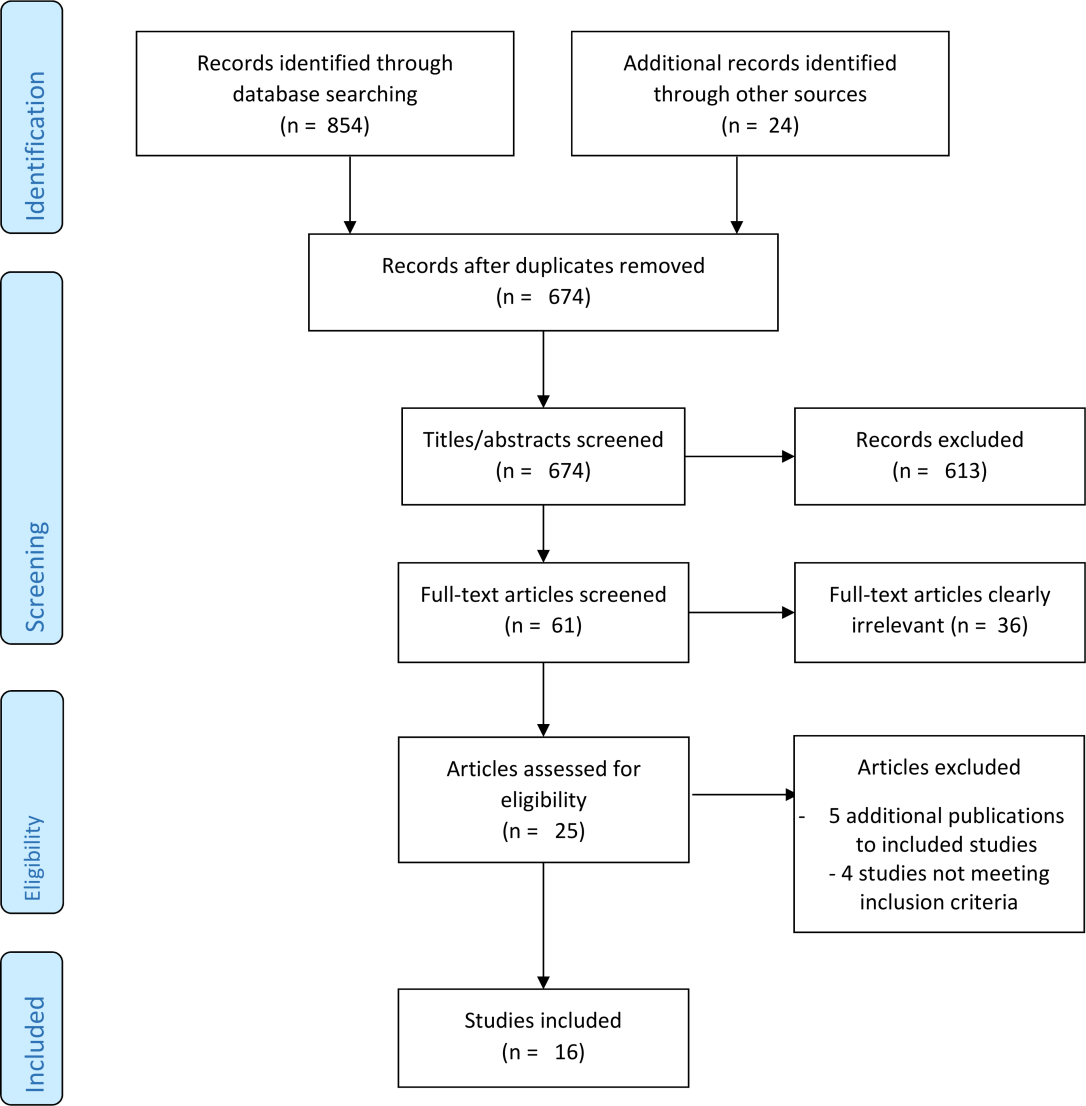
Flow diagram of the study selection process.

Our literature search identified 674 unique hits (see Fig 1 for a flow chart). Twenty-five potentially relevant articles were formally checked against inclusion criteria. Nine articles were excluded: five [16–20] reported additional findings or protocols of included studies not providing information relevant to our review, two did not have GPs as an identifiable subgroup [21,22], one was a qualitative study [23], and one did not address actual placebo use but a hypothetic case [24].

### Characteristics of included studies

We included 16 studies (reported in 16 publications) for which data on placebo use by GPs were available [5–7,10,11,25–35]. The 16 studies were published between 2003 and 2017 and had been performed in 13 different countries (Table 1). Eight studies used high-quality sampling strategies [5–7,10,28–30,33], the remaining eight used less adequate or unclear sampling methods. Response rates ranged from 16% to 79% in studies with high-quality sampling and from 8% to 100% in studies with suboptimal sampling methods. Only one study was rated high quality both regarding for sampling and response rate [29] (see S1 Table for the full rating of all studies). The number of participating GPs was much higher in studies with high quality sampling (2,471 in total, range 165 to 783; compared to 520 in total, range 27 to 157). Eight studies also surveyed physicians from other medical specialties. Definitions of placebo interventions (see S2 Table), topics covered, wording of questions, and answer options varied greatly among included studies creating considerable difficulties for consistent data extraction. Definitions either included non-specific therapies or separated pure placebos and non-specific therapies, but no study exclusively focused on pure placebos. Eleven studies presented data on reasons for prescribing placebo interventions (S3 Table). In the four studies offering this answer option, 48% to 79% of GPs ticked that they hoped to elicit placebo or psychological effects. A larger number of studies included a variety of answer options related to patient expectations and demands, e.g. to calm the patient (agreement between 21% and 58%; 8 studies), to avoid conflict (29% to 70%; 2 studies), or to handle an unjustified demand of a patient (9% to 47%; 8 studies). A third group of answer options covered “medical” reasons, e.g. treating non-specific complaints (22% to 61%; 10 studies), use as a supplement to other therapies (16% to 54%; 7 studies) or using placebo treatment as a diagnostic tool (13% to 60%; 10 studies).

**Table 1:** Characteristics of included studies (2,911 participating GPs).

First author year [reference]	Country	Population	Sampling	Response rate	n GPs	Other disciplines	Additional information from authors	Number outcomes reported
Babel 2012 [25]	Poland	Southeast Poland	Convenience	Unclear	41	Int, Ped	Q, raw data subgroups	3 / 4
Babel 2013 [26]	Poland	Southeast Poland	Convenience	78%*	50	Int, Ped	Q, raw data subgroups	3 / 6
Braga-Simoes 2017 [27]	Portugal	Small region (Matosinhos)	All	74%	93	-	Not contacted	4 / 0
Fässler 2009 [28]	Switzerland	Regional (Canton Zurich)	Random	47%*	166	Ped	Q, subgroup data GP	3 / 2
Fässler 2011 [29]	Switzerland	Regional (Canton Zurich)	Random	79%	232	-	Q, additional analyses	1 / 0
Ferentzi 2011 [30]	Hungary	Country-wide	Random	16%	169	-	Q	2 / 8
Harris 2015 [31]	Canada	Academic, country-wide	Convenience online	8%*	42	Any	subgroup data GP	1 / 7
Holt 2009 [32]	New Zealand	Small region	Convenience	Unclear	157	-	No addition. information	4 / 6
Howick 2013 [6]	UK	Country-wide	Random	46%	783	-	Q, raw data	12 / 6
Hrobjartsson 2003 [10]	Denmark	Country-wide	Random	64%	182	Spec, Hosp	Not contacted	3 / 4
Kermen 2010 [5]	USA	Country-wide	Random	43%	412	-	Could not be contacted	3 / 7
Khan 2015 [35]	Pakistan	One city (Faisalabad)	Convenience	92%	80	Gyn, Ped, MO	Q	1 / 0
Linde 2014 [7]	Germany	Country-wide	Random	46%	319	Int, Ortho	Raw data	12 / 8
Meissner 2012 [33]	Germany	Regional (Bavaria)	Random	55%	208	-	Raw data	12 / 6
Nitzan 2004 [11]	Israel	Unclear	Convenience	67%	27	Hosp, Nurses	Did not reply	1 / 0
Shah 2009 [34]	India	One city (Ahmedabad)	Convenience	100%	30	Hosp, Resid	Did not reply	3 / 0

Number of outcome: the first figure indicates the number of frequency outcomes included in meta-analyses (maximum 12) / the second figure the number of specific intervention outcomes (maximum 11)

*only response rates across disciplines available

GP = general practitioners; Gy = gynecologists; Int = internal medicine; MO = medical officers; Ped = pediatrics; Spec = specialists in private practice; Hosp = hospital physicians; Ortho = orthopedists in private practice; Resid = resident doctors; Q = provided unpublished questionnaire

Four (out of a total of seven) studies including also other medical specialties did not report GP findings separately in the publication, but on request authors either provided subgroup analyses [28,31] or relevant raw data for re-analysis [25,26]. Full raw data for re-analysis was available for further three studies [6,7,33].

### Frequency of placebo use

**Fig 2:**
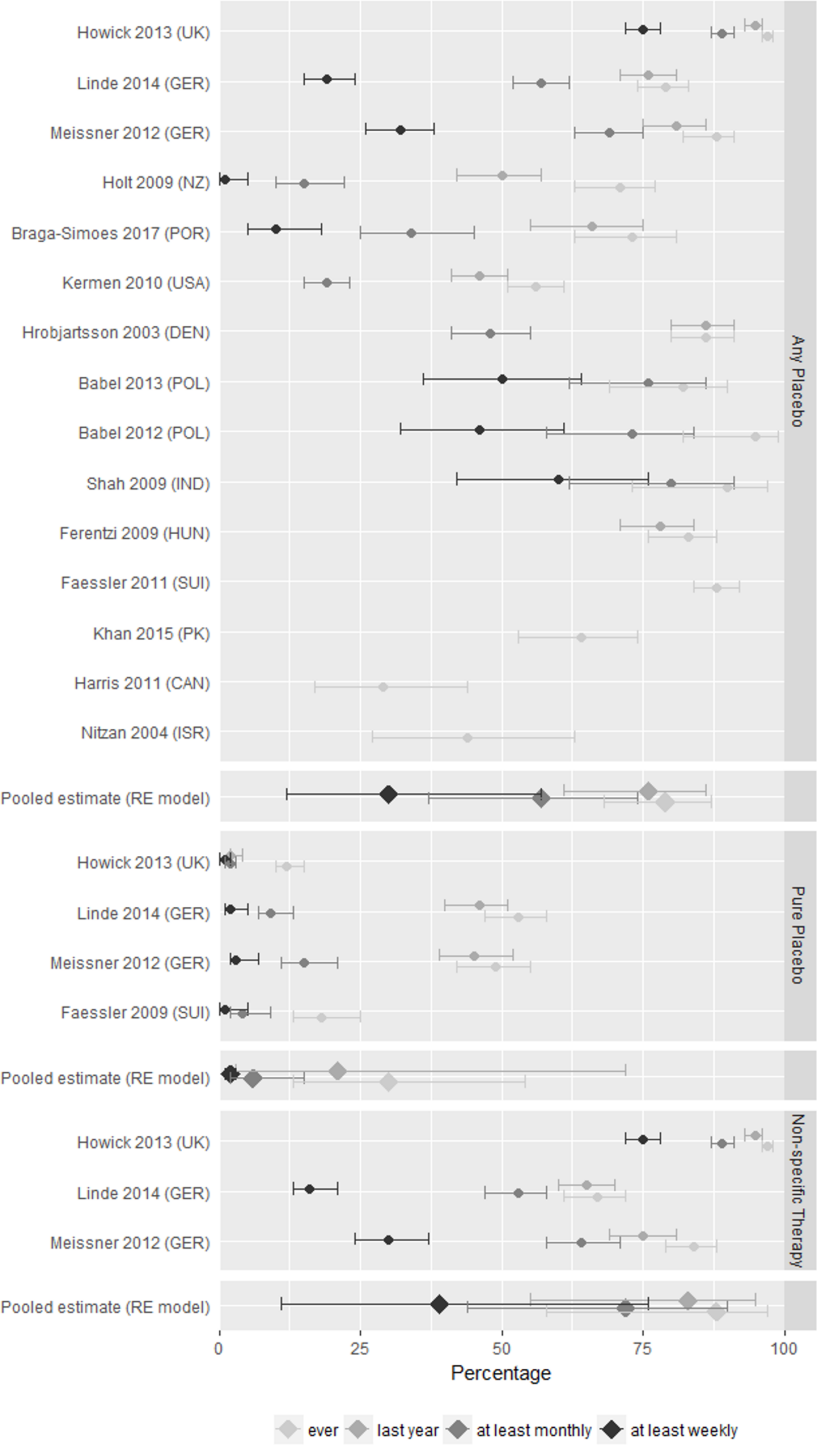
Percentages (95% confidence intervals) of GPs having used a placebo intervention (upper part), a pure placebo or a non-specific therapy (lower part) at least once in their career (light grey), last year (grey), using it at least monthly (dark grey) or at least weekly (black). RE = random effects.

Four studies reported data on only one of our 12 outcomes addressing frequency of placebo use and further nine studies on two to four outcomes. For three studies with a total of 1,310 GPs with available raw data, all 12 outcomes could be calculated. The reported use of any form of placebo or non-specific therapies varied much more than it could have been expected by chance alone. The percentage of GPs having used any form of placebo at least once in their career ranged from 29% to 97% (13 studies), of those having used it at least once in the last year from 46% to 95% (8 studies), at least monthly from 15% to 89% (10 studies), and at least weekly from 1% to 75% (8 studies; see upper parts of Fig 2 and S4 Table). Pooled random estimates were 79% (95%CI 68% to 87%), 76% (61% to 86%), 57% (37% to 74%), and 30% (12% to 57%), respectively. However, due to the pronounced heterogeneity (I^2^ between 96% and 99%), pooled estimates need to be interpreted with great caution. Four studies provided data on the use of pure placebos and three on the use of non-specific therapies separately, with three studies investigating both (Fig 2 and S4 Table, lower parts). The use of non-specific therapies by far outnumbered the use of pure placebo, but again estimates of usage varied strongly between studies. For example, the proportion of GPs with use at least monthly varied between 2% and 15% for pure placebos and between 53% and 89% for non-specific therapies, with use at least weekly between 1% and 3% for pure placebos and between 16% and 75% for non-specific therapies.

### Interventions used as placebos

**Fig 3:**
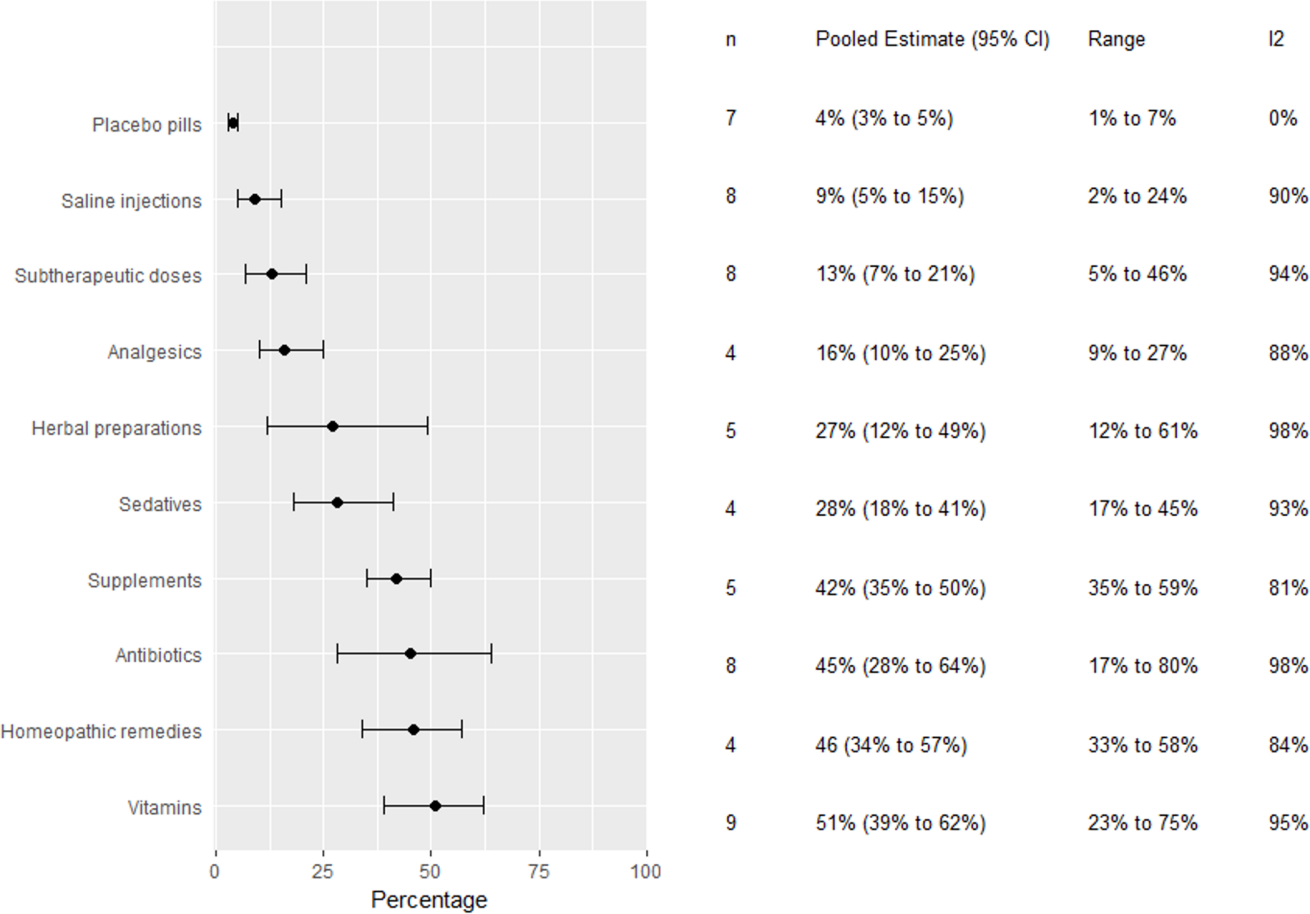
Percentage of GPs having use a defined intervention at least once as a placebo. Graphically presented values are pooled estimates (95% confidence intervals) n = number of studies in which the intervention was investigated.

Eleven studies reported which interventions GPs used as pure placebos or non-specific therapies at least once in their career (see Fig 3). Again, the use of non-specific interventions by far outperformed the use of pure placebos, but the percentage of users varied greatly across studies. Saline injections had been used by 2% to 24% of GPs and placebo pills by 3% to 7%. Instead, use of vitamins was reported by 23% to 75% of GPs, of homeopathic remedies by 33% to 58%, of antibiotics by 17% to 69%, and of supplements by 35% to 59%. With the exception of the use of placebo pills (I^2^ = 0%) study findings varied much more than expected by chance alone (all I^2^ > 80%; see S5 Table providing findings of individual studies and details of meta-analyses).

### Comparison with other medical specialties

Seven studies provided data for our comparative analysis of use of any placebo interventions between disciplines (Table 2). Three studies compared GPs with specialists in private practice. The two larger studies with high quality sampling found a statistically significantly higher prevalence of placebo use among GPs while the third smaller study with low quality sampling did not. Three studies compared GPs and hospital physicians. Results differed strongly with one larger study with high quality sampling finding higher placebo use among GPs and the two smaller studies finding no differences. Three small studies compared placebo use among GPs with that of pediatricians and two with internists providing primary care. Differences were not statistically significant.

**Table 2:** Placebo use (ever of of any form of placebo) among GP compared to other disciplines.

First author Year (Country)	N use	N no use	N use	N no use	OR (95%CI)
	GPs		Specialists private practice		
Hrobjartsson 2003 (DEN)	157	25	56	80	8.97 (5.21, 15.44)
Linde 2014 (GER)	252	67	327	289	3.32 (2.43, 4.54)
Khan 2015 (PK)	51	29	64	30	0.82 (0.44, 1.55)
Pooled (RE)					2.94 (0.77, 11.15)
Heterogeneity: Q = 31.71, df = 2, p = <0.01, I^2^ = 96%					
	GPs		Hospital physicians		
Hrobjartsson 2003 (DEN)	157	25	100	85	5.34 (3.20, 8.90)
Nitzan 2004 (ISR)	12	15	19	12	0.51 (0.18, 1.44)
Shah 2009 (IND)	27	3	53	7	1.19 (0.28, 4.97)
Pooled					1.59 (0.37, 6.78)
Heterogeneity: Q = 17.6, df = 2, p<0.01, I^2^ = 85%					
	GPs		Pediatricians (primary care)		
Babel 2012 (POL)	39	2	46	8	3.39 (0.68, 16.92)
Babel 2013 (POL)	41	9	34	14	1.88 (0.72, 4.86)
Khan 2015 (PK)	51	29	48	16	0.59 (0.28, 1.21)
Pooled					2.19 (0.96, 4.97)
Heterogeneity: Q = 5.94, df = 2, p = 0.05, I^2^ = 66%					
	GPs		Internists (primary care)		
Babel 2012 (POL)	39	2	78	17	4.25 (0.93, 19.33)
Babel 2013 (POL)	41	9	60	13	0.99 (0.39, 2.52)
Pooled					1.81 (0.44, 7.39)
Heterogeneity: Q = 2.58, df = 1, p = 0.11, I^2^ = 61%					

OR = odds ratio; CI = confidence interval

## Discussion

Despite highly variable methods and prevalence estimates, the available surveys from a total of twelve countries provide clear evidence that many GPs use interventions which are considered placebos in the studies included. Only few (but comparably large) studies provide frequency estimates on pure placebos and non-specific therapies separately, but together with the data on which defined interventions have been used as placebos it is obvious that non-specific therapies are used far more often than pure placebos. The data suggest that frequency and patterns of usage vary considerably between countries. Only few studies compare placebo prescription across medical specialties. The available findings are inconsistent and inconclusive.

This is the first systematic review and meta-analysis of the use of placebo interventions among GPs. Based on the experiences from a broader qualitative review of empirical studies of placebo use in clinical practice published in the year 2010 [4] and thanks to the great cooperation of many of the survey authors who provided original questionnaires (and, if necessary, help with translation), additional analyses or raw data, we were able to compile reasonably comparable data from a quite diverse set of studies. When the literature search for the 2010 review was completed, only three [10,11,28] of the 16 studies now included had been available. A systematic analysis of the frequency of placebo use had not been performed.

In the past, the publication of placebo surveys among physicians has repeatedly triggered sensational reports in mass media. For example, based on a survey among US internists published in the British Medical Journal [12] on October 23, 2008, the New York Times published an article titled “Half of Doctors Routinely Prescribe Placebos” [36]. Articles with a similar message were published in the mass media after surveys among GPs. Do the data compiled in our review really justify the interpretation of such broad placebo use?

According to a survey in eleven countries [37,38], primary care physicians on average see between 96 (USA as country with the lowest estimate) and 242 (Germany as country with the highest estimate) patients per week, with GPs in the UK seeing 130 patients per week. Based on these figures, we roughly estimate that GPs have about 500 patient contacts per month in the UK and 1,000 in Germany. A combination of these figures with the findings of our review would mean that 2% of UK GPs use a pure placebo in at least 1 of 500 patient contacts [6], and that 9% to 15% of German GPs do so in at least 1 of 1,000 patient contacts [7,33]. This can hardly be considered frequent use. What seems to be used “routinely” by many GPs are non-specific therapies. Using the same approximation as for pure placebo, we estimate that 89% of GPs in the UK use a non-specific therapy in at least 1 of 500 patient contacts, and that between 57% and 69% of German GPs do so in at least 1 of 1,000 patient contacts.

However, is it really adequate to interpret the widespread use of non-specific therapies as use of placebos? The main argument for doing so is nicely summarized by Miller and Colloca: “… what makes substances or interventions count as placebos is the lack of specific efficacy in treating a specific patient’s condition based on the inherent properties of the treatment” [39]. However, there are several problems with this view. First, when hearing the word ‘placebo’ both physicians [16,23] and patients [29] usually intuitively think of pure placebos or words attributed to pure placebos (such as “inert”). As a consequence, many lay persons and physicians are surprised when they hear about the high prevalence of “placebo use”. Second, even from the perspective of research, it is not always clear whether a treatment has “specific efficacy” or not. For patients and practicing physicians, own beliefs and experiences strongly influence what is considered specifically active or not. For example, among German physicians the belief whether treatments such as acupuncture, homeopathy, herbs or osteopathy have effects over placebo varies strongly [40]. It seems very likely that subjective views and the wording of questions and answer options strongly influence survey findings on the use of non-specific therapies [4,8,9]. This is related to the third challenge. In our view, placebo researchers often misinterpret why physicians prescribe placebos in general and non-specific treatments in particular. Most researchers administering surveys assume implicitly or even explicitly that physicians use “placebos” mainly for eliciting placebo effects. For example, in the BMJ 2008 survey, the authors state that “placebo treatments include … physiologically active agents, such as vitamins or antibiotics, that the physicians prescribe solely or primarily to promote positive psychological effects” [12]. Only few respondents in the quantitative surveys actively argue against such definitions (but some do; see, for example [16]). However, there are reasons to assume that promoting placebo effects is more often an a posteriori justification than an a priori reason. For example, when checking qualitative studies on reasons for antibiotic prescribing in viral infections–a typical example for an impure placebo in surveys—included in a systematic review [41], we could not find a single study mentioning the word placebo or a related motive. Reported important reasons for antibiotic prescribing are perceived pressure by patients, lack of time, preserving an unstable patient-physician relationship, or diagnostic uncertainty. Qualitative studies on “irrational” [42], “nonscientific” [43] or otherwise difficult prescribing [44,45] suggest that the use of non-specific therapies is a strategy to deal with a variety of challenges in busy routine practice. Most of the reasons for placebo use reported in the studies included in our review strengthen this interpretation. Due to its conceptual problems Louhiala et al. consider the concept of impure placebos (which we call non-specific therapies in this paper) as useless, scientifically unsound and potentially harmful [9].

Hrobjartsson has argued that in the majority of cases physicians use (mostly impure) placebos for “convenience”, either to get a patient stop complaining or to avoid conflict [1]. Instead, Louhiala et al. believe that at least some of what in surveys is called impure placebo (for example, positive suggestions) actually can be considered “good doctoring” [9]. We are aware that the term non-specific therapies which we use in this review suffers from similar conceptual problems as the term impure placebo. But at least, it greatly reduces the risk of a simplistic interpretation of a complex phenomenon.

Currently, there is considerable interest in the prescription of (pure) placebos without deception (sometimes referred to as “open-label placebo” [46]). The idea is that patients are informed about the placebo effect and, after giving informed consent, openly receive an inert intervention. This is a fascinating approach respecting the autonomy of the patient and the professional integrity of the physician. The (few) available randomized trials suggest it can be feasible and effective [47]. It is clearly desirable to test and possibly implement this strategy on a larger scale in general practice. However, there might be a long way to go as placebo without deception might not be a solution to many of the problems in which GPs currently use non-specific therapies (e.g., when a patient with a cold demands an antibiotic).

Due to diversity of the studies included in our review regarding main focus, definitions, questionnaires, sampling methods, response rates, sample sizes and other quality issues the frequency estimates found in our review should only be considered crude indicators. The statistical heterogeneity of almost all of our pooled estimates is very high, but given the limited number of studies contributing to meta-analyses, problems in reliably operationalizing potential confounders, and the unclear interactions between the confounders we refrained from performing meta-regression analyses. Based on the data it seems likely that the use of placebos varies between countries regarding what is actually done (e.g., more German GPs seem to use pure placebos than GPs from the UK), but it also seems plausible that there are cultural differences regarding what is considered a non-specific therapy (e.g., GPs from the UK may follow more what is evidence-based, while German GPs may have a stronger belief in their own experience). Furthermore, it is likely that definitions and methods of asking make a difference. For example, one of the included studies [25] showed that using the term “non-specific therapies” instead of “placebo” was associated with higher placebo use estimates although the definition of the terms was exactly the same. Also, we assume that asking to rate a long list of examples of non-specific therapies (as in the large UK survey [6]) is associated with higher estimates than a global question. Finally, it is also possible that selection bias differs between countries.

In conclusion, high prevalence estimates of placebo use among GPs are primarily driven by the frequent use of non-specific therapies; pure placebos are used rarely. It seems likely that in the majority of cases non-specific therapies are not primarily used to elicit placebo effects but to cope with difficult situations in busy routine practice. In the view of the authors it can be misleading to subsume the use of non-specific therapies simply as ‘placebo use’. The issue of placebo and non-specific interventions should be addressed in GP training programs. Future research is needed to better understand why many GPs are using such interventions. Also, investigations should be conducted about how GPs, who do not use such strategies (or claim to do so), cope with the challenges in busy daily practice. We believe that such research primarily should be qualitative. International, large-scale surveys are needed to be able to directly compare the differences in placebo prescription behavior of GPs (and across medical disciplines) between countries. However, such surveys should carefully consider the major problems of the concept of impure placebos/non-specific therapies in the planning phase, avoid suggestive wording in questionnaires, and carefully discuss the limitations of the findings for avoiding simplistic interpretations in the mass media.

## Supporting information

S1 File Protocol. Protocol.
(PDF)

S2 File Search strategies. Search strategies for PubMed, Medline, Scopus and Google Scholar.
(PDF)

S3 File Raw data for meta-analysis. Raw data for meta-analysis.
(XLSX)

S4 File PRISMA checklist. (DOC)

S1 Table Results of quality assessment. Results of quality assessment of individual studies.
(PDF)

S2 Table Definitions. Definitions and examples of placebo interventions given to survey participants in included studies.
(PDF)

S3 Table Reasons. Reasons for prescribing placebo interventions.
(PDF)

S4 Table Numeric data on frequency of use. Proportion (95%CI) of physicians using any type of placebo, pure placebos and non-specific therapies.
(PDF)

S5 Table Specific interventions used as placebos. Proportions (95%CI) of physicians having used specific interventions as placebos.
(PDF)
