# Supplementary material for: How often do general practitioners use placebos and non-specific interventions? Systematic review and meta-analysis of surveys
Source: PLoS One. 2018 Aug 24;13(8):e0202211. doi: 10.1371/journal.pone.0202211 (PMC6108457; doi:10.1371/journal.pone.0202211)
Supplement: S1 File — Protocol. (PDF) [file pone.0202211.s001.pdf]

S1 File. Protocol

TECHNISCHE UNIVERSITÄT MÜNCHEN
Klinikum rechts der Isar, Institut für Allgemeinmedizin
Univ. Prof. Dr. Antonius Schneider

Protocol

Use of Placebo Interventions by General Practitioners

–

Systematic Review of Surveys

Version 30 March 2017 amended 4 August 2017

Klaus Linde[1], Oxana Atmann[1], Karin Meissner[2,3], Antonius Schneider[1], Ramona Meister[4], Levente Kriston[4], Christoph Werner[1]

[1]Institute of General Practice, Technical University of Munich, Orleansstr. 47,81667 München, Germany

[2]Fakultät Soziale Arbeit und Gesundheit, Hochschule für angewandte Wissenschaften Coburg, Friedrich-Streib-Straße 2, D-96450 Coburg, Germany

[3]Institute of Medical Psychology, Ludwig-Maximilians-University Munich, Goethestr. 31, 80336 München, Germany

[4]Department of Medical Psychology, University Medical Center Hamburg-Eppendorf, Germany

S1 File. Protocol

1. Background

The use of placebo interventions in clinical practice is ethically, professionally and legally controversial. In this situation it is important to know about the frequency and circumstances of placebo use. In 2010 a part of our group (KL, AS) were involved in a systematic review summarizing the available cross-sectional surveys on the use of placebo interventions in clinical practice, on the respective motivations, and on attitudes of health care professionals, students, and patients towards placebo use [1]. Since the completion of the literature search for this review a number of new high quality surveys, mainly performed in primary care providers, have been published (e.g. [2-4]). Compared to other specialties, the use of non-specific interventions or impure placebos seems to be particularly widespread among GPs [1,5]. This is plausible when considering that GPs see many patients in which it is difficult to make a distinct diagnosis [6] and who have minor ailments [7].

We aim to perform a new systematic review including – if possible – also a meta-analysis of cross-sectional surveys to investigate the use of placebo interventions among general practitioners.

2. Definitions of terms used in this protocol

General practitioners (GPs): General practitioners, family medicine physicians and similar specialists of general practice/family medicine.

Placebo intervention: Any intervention considered a placebo (pure or impure) in a primary study.

Pure placebos: 1. Products such as placebo tablets or pure placebo pills without active agent and manufactured to be a placebo intervention; 2. Saline injections or infusions provided as placebos; other clearly inert interventions provided by GP as a placebo (i.e., considered as inert by the prescribing GP).

Non-specific therapies (= impure placebo): Placebo interventions other than pure placebos, e.g. antibiotics in viral infections not considered indicated by the provider.

3. Specific Objectives

Our primary objective is:
To estimate the proportion of GPs who have used A) any type of placebo intervention (pure or impure), B) pure placebos, and C) a non-specific therapies.

Secondary objectives are:

1. To estimate the proportion of GPs who have used defined placebo interventions (sugar tablets and similar products, NaCl injections or infusions for placebo purposes, non-indicated antibiotics, other treatments reported in at least three primary studies).

2. To compare the proportion of GPs who have used A) any placebo interventions, B) pure placebos, and C) non-specific therapy (operationalized as for the primary objective) with the proportion of those of other medical disciplines who have used these interventions.

4. Selection criteria

Year of publication: To be included, studies must have been published in the year 2000 or later and data collection must have taken place 1997 or later. Studies published before 2003 for which information when data collection took place cannot be obtained, but in which there is no hint that data collection took place before 1997, will be included. Rationale: Older studies are less likely to represent current placebo use.

Study design: To be included studies must be cross-sectional quantitative surveys (studies using a standardized questionnaire in a sample of physicians at a given point in time or in a short time period).

Study participants: To be included at least one group of at least 25 study participants must be GPs for which the following criterion regarding outcomes is met. Studies in mixed physician populations with more than 80% of participants being GPs will be included. Studies in “primary care providers” including less than 80% GPs, but including a group of at least 25 GPs, will be included on a preliminary level. We will try to obtain subgroup data for GP from authors; if such data cannot be obtained we will decide on inclusion on rules that have to be determined in an amendment.

Outcomes: Studies will be included if the numerical data on any of the following outcomes can be obtained (either form the report or upon request from the authors, or by re-analysis of raw data): Proportion of GPs who have used a placebo intervention, a pure placebo, or a non-specific therapy ever in their career at least once, in the last 12 months at least once, at least monthly, or at least weekly.

5. Literature search

For our previous systematic review on placebo use in medicine in general [1] a comprehensive collection of placebo surveys until 2009 was established. Since then the literature on the topic was followed semi-systematically (repeated PubMed searches combining “Placebo[ti]” and “survey” and Citation checks of key publications). Therefore, a considerable collection of relevant studies was available in house when starting the project.

The main electronic literature searches were performed on February 21 and 22, 2017 in PubMed, Medline (using Web of Science) and Scopus. PubMed was searched using a strategy combining a subject term (placebo in title), a combination of design terms (survey and related terms) and a combination of field terms (general practice an related terms). As this strategy failed to identify two relevant surveys and additional Medline search focusing mainly on title words (excluding publications likely to be placebo-controlled trials) was performed. Scopus was searched using a similar strategy as for Medline. All search strategies and hits were saved. All citations were imported into Endnote software. Duplicates were then eliminated. Monthly update searches will be performed for PubMed and Medline.

In addition citations searches were done in Google Scholar for four key publications [1,5,8,9]. Only possibly relevant articles identified were imported into Endnote (see also selection process).

6. Selection process

At least two reviewers will screen independently search hits (titles and abstracts) from the main electronic search for potentially eligible publications. Clearly irrelevant search hits are excluded. Publications considered potentially eligible by at least one reviewer will be obtained (unless they are already available) in full text. The screening process will be documented in Endnote.

Publications citing key articles identified through Google Scholar will be screened by a single reviewer (KL). Only publications for which full text articles will be obtained are imported into Endnote. Potentially relevant articles identified by other means will be entered into Endnote directly.

All potentially relevant full text publications will be checked formally against the selection criteria by at least two reviewers using a form (see appendix). Disagreement will be resolved by discussion and documented. Reasons for exclusions also will be documented.

7. Data extraction

Information on bibliometric source, country of the survey, participants, definitions, methods and findings will be extracted using a pretested form (see appendix) by at least two reviewers independently. Disagreement will be resolved by discussion and documented. Whenever possible we will obtain the questionnaires used in the studies.

In particular, we will extract the following data relevant to our objective:

- number of physicians analyzed (line C on page 2 of the extraction form)
- number of physicians having used any type placebo intervention at least once in their career, in the last 12 months at least once, at least monthly, and at least weekly (lines D to G);
- number of physicians having used a pure at least once in their career, in the last 12 months at least once, at least monthly, and at least weekly (lines H to K);
- number of physicians having used a non-specific at least once in their career, in the last 12 months at least once, at least monthly, and at least weekly (lines L to O).
- number of physicians having used defined placebo interventions (sugar tablets and similar products, NaCl injections or infusions for placebo purposes, non-indicated antibiotics, other treatments reported in at least three primary studies) in their career (lines P to Z);
- If data for other physician groups beside GPs are reported these data will be extracted for all groups.

The list of specific types of placebos to extract was based on screening the types reported available studies likely to meet selection criteria. If a specific type of placebo was reported in at least two studies it was included in the list.

If included publications or reports do not report the outcomes listed above although they were measured or probably measured we will contact the authors and try to obtain these data.

8. Quality assessment

We checked the criteria in a widely used tool for observational studies (The Newcastle-Ottawa Scale, http://www.ohri.ca/programs/clinical_epidemiology/oxford.asp) and the quality

assessment used in a recent major meta-analysis of cross-sectional surveys [10]. Based on a review of these instruments we decided to assess the following issues:

1. Was the underlying population adequately defined?
   Answer options: Yes - e.g. “all registered GPs in Wales”. No - e.g., “we invited GPs participating in three CME events to participate”.
2. Was the procedure to draw a sample from the population adequate?
   Answer options: Yes - random sampling or equivalent methods (e.g. all GPs in a certain area were invited). No - any methods unlikely or uncertain to result in a representative sample; the answer is also no if the answer to question 1 is no.
3. Is the response rate sufficiently high to rule out selection bias?
   Answer options: Yes - >70% of those successfully contacted participated. Uncertain - 40 to 70% participated. No - <40% or no response rate available.
4. Did more than 200 GPs participate?
   Answer options: Yes/No
5. Was there some systematic pre-testing or validation of the questionnaire?
   Answer options: Yes - e.g. a quantitative pre-test, qualitative interviews etc. No
6. Were participants described?
   Answer options: Yes - age, gender and one additional information (e.g. practice experience, practice location etc.) described. No.

9. Data analysis (added after data extraction but before analysis on 4 August 2017)

9.1. Summarizing results of single studies and data entry into an MS Excel data file

As described above all outcome data are extracted as absolute frequencies (number of physicians meeting a defined criterion). For obtaining proportions, these numbers are divided by the number of participants. Missing answers will be counted as not meeting the criterion. Proportions will be transformed into logits (logarithmic odds) and the respective standard errors and 95% confidence intervals will be calculated for all studies and outcomes. The logits will be used for meat-analyses and transformed back into proportions afterwards [11]. Small study bias will be assessed by visual investigation of funnel plots

9.2. Meta-analysis on frequency of placebo use and use of specific types of placebos

Our 12 (3 types of placebos x 4 indicators of frequency of use) outcomes of primary interest are the proportions of GPs having used any placebo, pure placebos or non-specific therapies intervention at least once in their career, at least once last in the last year, at least monthly or at least weekly. Outcomes of secondary interest are the specific types of placebos.

Statistical analyses will be performed according to actual guidelines [12]. We will conduct random-effects meta-analyses for each outcome using the restricted maximum likelihood estimator. The extent of statistical heterogeneity will be tested for significance using Cochrane´s Q-test and quantified by means of the I^2 statistic. We assume that the use of placebos may vary between countries regarding what is actually done (e.g., German GPs may use more pure placebos than GPs from the UK) and regarding what is considered a non-specific therapy (e.g., GPs from UK may follow more what is evidence-based, while German GPs may have a stronger belief in their own experience). Furthermore, we assume that the method of asking may make a difference (e.g., using the term non-specific therapy may result in higher use estimates than the term placebo). Finally, it is possible that the influence of

selection biases differs between countries. However, given the low number of studies contributing to meta-analyses, problems in reliably operationalizing these variables, and the unclear interplay between the variables we refrain from meta-regression or subgroup analyses for country, and method of asking. Results will be visually displayed as forest plots. In these forest plots we will group studies according to the adequacy of the sampling method. However, as the eight studies (of 15 studies altogether) using high-quality sampling include about 85% of all participating GPs we will not calculate separate pooled estimates in the main analysis (this will be done in an additional sensitivity analysis).

9.3. Comparison of placebo use among GPs and other medical disciplines

Based on the limited available data only the use of any placebo at any time in the career will be compared between GPs, specialists in private practice, hospital physicians, pediatricians, and internists providing primary care will be analysed. All comparisons are direct (comparison of GPs and the other discipline in the same survey). In each study comparing GPs to other groups, odds ratios with 95% confidence intervals will be calculated from the two proportions. The analyses will be performed on the log-scale and the meta-analytic results will be transformed back to the odds ratio scale for interpretation.

All analyses will be performed in the open source statistical environment R with the package metaphor [13].

10. Amendments

Changes (which go beyond minor re-wording of the text without changing contents or processes) and additions made to the protocol from 30 March 2017 for this update are listed and explained in the Appendix Table.

11. References

1. Fässler M, Meissner K, Schneider A, Linde K. Frequency and circumstances of placebo use in clinical practice--a systematic review of empirical studies. BMC Med 2010, 8:15.
2. Meissner K, Höfner L, Fässler M, Linde K. Widespread use of pure and impure placebo interventions by GPs in Germany. Fam Pract 2012;29:79-85.
3. Howick J, Bishop FL, Heneghan C, Wolstenholme J, Stevens S, Hobbs FD, Lewith G. Placebo use in the United kingdom: results from a national survey of primary care practitioners. PLoS One. 2013;8:e58247.
4. Kermen R, Hickner J, Brody H, Hasham I: Family physicians be lieve the placebo effect is therapeutic but often use real drugs as placebos. Fam Med 2010, 42:636-642.
5. Hrobjartsson A, Norup M: The use of placebo interventions in medical practice- a national questionnaire survey of Danish clinicians. Eval Health Prof 2003, 26:153-165.

6. O'Riordan M, Dahinden A, Aktürk Z, Ortiz JM, Dağdeviren N, Elwyn G, Micallef A, Murtonen M, Samuelson M, Struk P, Tayar D, Thesen J. Dealing with uncertainty in general practice: an essential skill for the general practitioner. Qual Prim Care. 2011;19:175-81.
7. Morris CJ, Cantrill JA, Weiss MC. GPs attitudes to minor ailments. Fam Pract 2001;18: 581-5.
8. Nitzan U, Lichtenberg P. Questionnaire survey on use of placebo. BMJ. 2004 Oct 23;329(7472):944-6.
9. Tilburt JC, Emanuel EJ, Kaptchuk TJ, Curlin FA, Miller FG. Prescribing "placebo treatments": results of national survey of US internists and rheumatologists. BMJ. 2008 Oct 23;337:a1938. doi: 10.1136/bmj.a1938.
10. Rotenstein LS, Ramos MA, Torre M, Segal JB, Peluso MJ, Guille C, Sen S, Mata DA. Prevalence of depression, depressive symptoms, and suicidal ideation among medical students: a systematic review and meta-analysis. JAMA. 2016;316:2214-36.
11. Lipsey MW, Wilson DB. Practical Meta-Analysis. Thousand Oaks: Sage, 2001.
12. Higgins JPT, Green S, (Eds): Cochrane Handbook for Systematic Reviews of Interventions Version 5.1.0 [updated March 2011]. The Cochrane Collaboration; 2011.
13. Viechtbauer W. Conducting meta-analyses in R with the metafor package. J Stat Softw. 2010;36: 1–48.
14. Koch K, Miksch A, Schürmann C, Joos S, Sawicki PT: The German Health Care System in international comparison: the primary care physicians´ perspective. Dtsch Arztebl Int 2011; 108(15): 255–61.

S1 File. Protocol

Appendix Table

Changes and additions made to the original protocol from 30 March 2017 for the amended version from 4 August 2017

Section	30 March 2017	4 August 2017	Explanation
3. Specific objectives	Primary objective:<br>To estimate the proportion of GPs who have used<br>1) a placebo intervention in the last 12 months at least once;<br>2) a pure placebo in the last 12 months at least once;<br>3) a non-specific therapy in the last 12 months at least once.<br>Rationale for 12 months: this reflects current use and is a period relatively easy to memorize. Ever use will be also documented.<br>Secondary objective<br>1.To estimate the proportion of GPs who have used defined placebo interventions (sugar tablets and similar products, NaCl injections or infusions for placebo purposes, non-indicated antibiotics, other treatments reported in at least three primary studies) in the last 12 months at least once<br>2. To estimate among placebo users the frequency of the use of 1) placebo interventions; 2) pure placebos; 3) non-specific therapies in the last 12 months.<br>3. To compare the proportions of physicans who have used at least once 1) a placebo intervention; 2) a pure placebo; 3) a non-specific therapy (operationalized as for the primary objective) in the last 12 months among GPs, specialists in private practice and physicians in hospitals.	Our primary objective is:<br>To estimate the frequency of the use of A) any type of placebo intervention (pure or impure); B) pure placebos; C) a non-specific therapies among GPs<br><br>Secondary objectives are:<br>1. To estimate the proportion of GPs who have used defined placebo interventions (sugar tablets and similar products, NaCl injections or infusions for placebo purposes, non-indicated antibiotics, other treatments reported in at least three primary studies) at least once.<br>2. To compare the use of A) any placebo interventions, B) pure placebos, and C) non-specific therapy (operationalized as for the primary objective) among GPs with those of other medical disciplines.	During extraction it became clear that the number of physicians using placebos in the last 12 months could not be extracted at all or in a reliable manner in 7 of 15 studies. Many studies asked physicians to mark categories such as "weekly, monthly, less than monthly, never" and reported the respective frequencies. Furthermore, frequency of use was rarely reported with median numbers or similar descriptive summary statistics. Therefore, we decided to modify our objective to focusing on estimates of frequency based and what type of data was most often available.<br>For defined placebo interventions most studies only ask whether it was used at all. Due to the scarce data available we decided to only analyze ever use of any placebo for the comparison of medical disciplines
4. Selection criteria,	Outcomes: To be included in the final analyses (see 6.)	Outcomes: Studies will be included if the numerical data on any of the following	This re-wording describes the actual selection process

paragraph outcomes	numerical results for at least one of the following outcomes must have been reported or must have been obtained from authors on request: (Proportion of GPs who have used 1. a placebo intervention; 2. a pure placebo; 3. a non-specific therapy in the last 12 months at least once) OR (frequency of the use of 1. placebo interventions; 2. pure placebos; 3. non-specific therapies in the last 12 months). If it seems likely that a study measured one of the outcomes we include the study preliminarily and will try to obtain results from the authors.	outcomes can be obtained (either form the report or upon request from the authors, or by re-analysis of raw data): Proportion of GPs who have used a placebo intervention, a pure placebo, or a non-specific therapy ever in their career at least once, in the last 12 months at least once, at least monthly, or at least weekly.	reflecting the extraction problems with use in the last 12 months and frequency described above.
7. Data extraction, part on outcome extraction	In particular, we will extract the following outcome data:<br>Primary<br>1) number of participants having used a placebo intervention in the last 12 months at least once (divided by the total number of participants);<br>2) number of participants having used a pure placebo in the last 12 months at least once (divided by the total number of participants);<br>3) number of participants having used a non-specific therapy in the last 12 months at least once (divided by the total number of participants).<br>Secondary<br>4) number of participants having used defined placebo interventions (sugar tablets and similar products, NaCl injections or infusions for placebo purposes, non-indicated antibiotics, other treatments reported in at least three primary studies) in the last 12 months at least once;<br>5) among placebo users frequency data on the	In particular, we will extract the following data relevant to our objective:<br>• number of physicians analyzed (line C on page 2 of the extraction form)<br>• number of physicians having used any type placebo intervention at least once in their career, in the last 12 months at least once, at least monthly, and at least weekly (lines D to G);<br>• number of physicians having used a pure at least once in their career, in the last 12 months at least once, at least monthly, and at least weekly (lines H to K);<br>• number of physicians having used a non-specific at least once in their career, in the last 12 months at least once, at least monthly, and at	The final wording more accurately reflects the final outcome extraction strategy (operationalized in the final extraction form and the related instructions from 11 May 2017)

	use of 1) placebo interventions; 2) pure placebo; 3) non-specific therapies in the last 12 months.	least weekly (lines L to O).<br>• number of physicians having used defined placebo interventions (sugar tablets and similar products, NaCl injections or infusions for placebo purposes, non-indicated antibiotics, other treatments reported in at least three primary studies) in their career (lines P to Z);<br>• If data for other physician groups beside GPs are reported these data will be extracted for all groups.	
9. Data analysis	Only headlines and some text on interpretation issues for discussion	Analysis plan newly added. Interpretation issues deleted (not relevant for statistical analysis)	Details of the analysis could be planned only when having an overview of the data available.
