# Supplementary material for: How often do general practitioners use placebos and non-specific interventions? Systematic review and meta-analysis of surveys
Source: PLoS One. 2018 Aug 24;13(8):e0202211. doi: 10.1371/journal.pone.0202211 (PMC6108457; doi:10.1371/journal.pone.0202211)
Supplement: S1 Table — Results of quality assessment of individual studies. (PDF) [file pone.0202211.s005.pdf]

S1 Table. Results of quality assessment. Results of quality assessment of individual studies

Results of quality assessment of individual studies

Study	Item 1	Item 2	Item 3	Item 4	Item 5	Item 6
Babel 2012	no	no	unclear	no	no	yes
Babel 2013	no	no	2	no	no	yes
Braga-Simoes 2017	no	no	unclear	no	yes	yes
Fässler 2009	yes	yes	unclear	no	yes	yes
Fässler 2011	yes	yes	yes	yes	yes	no
Ferentzi 2009	yes	yes	no	no	no	yes
Harris 2011	no	no	no	no	no	no
Holt 2009	no	no	unclear	no	no	no
Howick 2013	yes	yes	unclear	yes	yes	yes
Hrobjartsson 2003	yes	yes	unclear	no	yes	no
Kermen 2010	yes	yes	unclear	yes	no	yes
Khan 2015	no	no	yes	no	no	no
Linde 2014	yes	yes	unclear	yes	yes	yes
Meissner 2012	yes	yes	unclear	yes	yes	yes
Nitzan 2004	no	no	unclear	no	no	yes
Shah 2009	no	no	unclear	no	no	no

Item 1: Was the underlying population adequately defined and relevant?
Item 2: Was the procedure to draw a sample from the population adequate?
Item 3: Is the response rate sufficiently high to rule out selection bias? (<40% = no: 40% to 70% = unclear; >70% = yes)
Item 4: Did more than 200 GPs participate?
Item 5: Was there some systematic pre-testing or validation of the questionnaire?
Item 6: Were participating GPs described?

5. S-Table 2 - numeric data on frequency of use

Proportion (95%CI) of physicians using any type of placebo, pure placebos and non-specific therapies

Study	N	Ever	Last year	≥ monthly	≥weekly
**Any Placebos**					
Babel 2012 (POL)	41	0.95 (0.82, 0.99)		0.73 (0.58, 0.84)	0.46 (0.32, 0.61)
Babel 2013 (POL)	50	0.82 (0.69, 0.90)		0.76 (0.62, 0.86)	0.50 (0.36, 0.64)
Braga-Simoes 2017 (POR)	93	0.73 (0.63, 0.81)	0.66 (0.55, 0.75)	0.34 (0.25, 0.45)	0.10 (0.05, 0.18)
Fässler 2011 (SUI)	232	0.88 (0.84, 0.92)			
Ferentzi 2009 (HUN)	169	0.83 (0.76, 0.83)	0.78 (0.71 0.84)		
Harris 2011 (CAN)	42	0.29 (0.17, 0.44)			
Holt 2009 (NZ)	157	0.71 (0.63, 0.77)	0.50 (0.42, 0.57)	0.15 (0.10, 0.22)	0.01 (0.00, 0.05)
Howick 2013 (UK)	783	0.97 (0.96, 0.98)	0.95 (0.93, 0.96)	0.89 (0.87, 0.91)	0.75 (0.72, 0.78)
Hrobjartsson 2003 (DEN)	182	0.86 (0.80, 0.91)	0.86 (0.80, 0.91)	0.48 (0.41, 0.55)	
Kermen 2010 (USA)	412	0.56 (0.51, 0.61)	0.46 (0.41, 0.51)	0.19 (0.15, 0.23)	
Khan 2015 (PK)	80	0.64 (0.53, 0.74)			
Linde 2014 (GER)	319	0.79 (0.74, 0.83)	0.76 (0.71, 0.81)	0.57 (0.52, 0.62)	0.19 (0.15, 0.24)
Meissner 2012 (GER)	208	0.88 (0.82, 0.91)	0.81 (0.75, 0.86)	0.69 (0.63, 0.75)	0.32 (0.26, 0.38)
Nitzan 2004 (ISR)	27	0.44 (0.27, 0.63)			
Shah 2009 (IND)	30	0.90 (0.73, 0.97)		0.80 (0.62, 0.91)	0.60 (0.42, 0.76)
Studies/participants		15/2555	8/2323	10/2275	8/1681
RE pooled estimate		0.79 (0.68, 0.87)	0.76 (0.61, 0.86)	0.57 (0.37, 0.74)	0.30 (0.12, 0.57)
Heterogeneity		Q=323, df=14, p<0.01, I²=96%	Q=340, df 7, p<0.01, I²=98%	Q=579, df=9, p<0.01, I²=98%	Q=380, df=7, p<0.01, I²99%
**Pure Placebos**					
Fässler 2009 (SUI)	166	0.18 (0.13, 0.25)		0.04 (0.02, 0.09)	0.01 (0.00, 0.05)
Howick 2013 (UK)	783	0.12 (0.10, 0.15)	0.02 (0.02, 0.04)	0.02 (0.01, 0.03)	0.01 (0.00, 0.02)
Linde 2014 (GER)	319	0.53 (0.47, 0.58)	0.46 (0.40, 0.61)	0.09 (0.07, 0.13)	0.02 (0.01, 0.05)
Meissner 2012 (GER)	208	0.49 (0.42, 0.55)	0.45 (0.39, 0.52)	0.15 (0.11, 0.21)	0.03 (0.02, 0.07)
Studies/participants		4/1476	3/1310	4/1476	4/1476
RE pooled estimate		0.30 (0.13, 0.54)	0.21 (0.03, 0.72)	0.06 (0.02, 0.15)	0.02 (0.01, 0.02)
Heterogeneity (I²)		Q=221, df=3, p<0.01; I²=98%	Q=201, df=2, p<0.01, I²=99%	53.7, df=3, p<0.01, I²=95%	Q=6.88, df=3; p=0.08, I²=57%
**Non-specific therapies**					
Howick 2013 (UK)	783	0.97 (0.96, 0.98)	0.95 (0.93, 0.96)	0.89 (0.87, 0.91)	0.75 (0.72, 0.78)
Linde 2014 (GER)	319	0.67 (0.61, 0.72)	0.65 (0.60, 0.70)	0.53 (0.47, 0.58)	0.16 (0.13, 0.21)
Meissner 2012 (GER)	208	0.84 (0.79, 0.88)	0.75 (0.69, 0.81)	0.64 (0.58, 0.71)	0.30 (0.24, 0.37)
Studies/participants		3/1310	3/1310	3/1310	3/1310
RE pooled estimate		0.88 (0.58, 0.97)	0.83 (0.55, 0.95)	0.72 (0.44, 0.90)	0.39 (0.11, 0.76)
Heterogeneity (I²)		Q=135, df=2, p<0.01, I²=99%	Q=134, df=2; p<0.01, I²=99%	Q=166, df=2, p<0.01, I²=99%	Q0309, df=2, p<0.01, I²=99%

n = number of studies with data

6. S-Table 3 - specific interventions used as placebos

Proportions (95%CI) of physicians having used specific interventions as placebos (part 1 – less frequently used interventions)

Study	n	Placebo Pill	NaCl	Subtherapeutic doses	Analgesics	Herbal preparations
Babel 2012 (POL)	41			0.05 (0.01, 0.18)		
Babel 2013 (POL)	50	0.01 (0.00, 0.14)	0.08 (0.03, 0.19)	0.16 (0.08, 0.29)		
Braga-Simoes 2017 (POR)	93					
Fässler 2009	166	0.05 (0.02, 0.09)	0.12 (0.08, 0.18)			
Fässler 2011 (SUI)	232					
Ferentzi 2009 (HUN)	169	0.04 (0.02, 0.08)	0.24 (0.18, 0.31)	0.12 (0.08, 0.18)	0.27 (0.21, 0.34)	
Harris 2011 (CAN)	42	0.07 (0.02, 0.20)	0.17 (0.08, 0.31)	0.12 (0.05, 0.26)	0.19 (0.10, 0.34)	0.21 (0.12, 0.36)
Holt 2009 (NZ)	157	0.02 (0.01, 0.06)	0.02 (0.01, 0.06)	0.09 (0.05, 0.26)		0.12 (0.08, 0.18)
Howick 2013 (UK)	783	0.04 (0.03, 0.06)	0.10 (0.08, 0.13)	0.46 (0.43, 0.49)		
Hrobjartsson 2003 (DEN)	182		0.05 (0.03, 0.09)			
Kermen 2010 (USA)	412	0.03 (0.02, 0.05)	0.06 (0.04, 0.09)	0.10 (0.07, 0.13)	0.09 (0.07, 0.12)	0.12 (0.09, 0.15)
Linde 2014 (GER)	319			0.08 (0.05, 0.11)	0.14 (0.10, 0.18)	0.42 (0.37, 0.48)
Meissner 2012 (GER)	208					0.61 (0.54, 0.67)
Nitzan 2004 (ISR)	27					
Shah 2009 (IND)	30					
Studies		7	8	8	4	5
RE pooled estimate		0.04 (0.03, 0.05)	0.09 (0.05, 0.15)	0.13 (0.07, 0.21)	0.16 (0.10, 0.25)	0.27 (0.12, 0.49)
Heterogeneity (I^2)		Q=5, df=6, p<0.440, I^2=0%	Q=56, df=7, p<0.01, I^2=90%	Q=290, df=7, p<0.01, I^2=94%	Q=29, df=3, p<0.01, I^2=88%	Q=180, df=4, p<0.01, I^2=98%

Appendix to
Linde et al. Frequency of placebo use among general practitioners

Proportions (95%CI) of physicians having used specific interventions as placebos (part 2 – more frequently used interventions)

Study	n	Sedatives	Supplements	Antibiotics	Homeopathic remedies	Vitamins
Babel 2012 (POL)	41		0.59 (0.43, 0.72)		0.41 (0.28, 0.57)	0.73 (0.58, 0.84)
Babel 2013 (POL)	50		0.36 (0.24, 0.50)		0.58 (0.44, 0.71)	0.60 (0.46, 0.73)
Braga-Simoes 2017 (POR)	93					
Fässler 2009	166					
Fässler 2011 (SUI)	232					
Ferentzi 2009 (HUN)	169	0.29 (0.23, 0.36)		0.17 (0.12, 0.24)		0.75 (0.68, 0.81)
Harris 2011 (CAN)	42			0.43 (0.29, 0.58)		0.48 (0.33, 0.62)
Holt 2009 (NZ)	157			0.69 (0.61, 0.76)		0.39 (0.32, 0.47)
Howick 2013 (UK)	783		0.39 (0.35, 0.42)	0.80 (0.77, 0.83)		
Hrobjartsson 2003 (DEN)	182	0.45 (0.38, 0.52)		0.70 (0.63, 0.76)		0.48 (0.41, 0.55)
Kermen 2010 (USA)	412			0.40 (0.35, 0.45)		0.23 (0.19, 0.27)
Linde 2014 (GER)	319	0.17 (0.13, 0.21)	0.35 (0.30, 0.41)	0.34 (0.29, 0.39)	0.33 (0.28, 0.39)	0.43 (0.37, 0.48)
Meissner 2012 (GER)	208	0.25 (0.20, 0.32)	0.49 (0.42, 0.55)	0.17 (0.12, 0.23)	0.52 (0.46, 0.59)	0.52 (0.45, 0.59)
Nitzan 2004 (ISR)	27					
Shah 2009 (IND)	30					
Studies		4	5	8	4	9
RE pooled estimate		0.28 (0.18, 0.41)	0.42 (0.35, 0.50)	0.45 (0.28, 0.64)	0.46 (0.34, 0.57)	0.51 (0.39, 0.62)
Heterogeneity (I^2)		Q=44, df=3, $p<0.01$, I^2=93%	Q=16, df=4, $p<0.01$, I^2=81%	Q=463, df=7, $p<0.01$, I^2=98%	Q=24, df=3, $p<0.01$, I^2=84%	Q=151, df=8, $p<0.01$, I^2=95%

7. **S-Table 4** - reasons for prescribing placebo interventions

Reasons for prescribing placebo interventions

Reason	Study: percentage agreement to reasons proposed in questionnaire
**Placebo/psycholoigcal effects**	
Eliciting placebo effects	Fässler 2009: 69%; Hrobjartsson: 48%
Possible psychological effect	Howick: 48% (NST)/51% (PP); Meissner: 79% (NST)/77% (PP)
**Group expectations and demands**	
Conform with requests of the patient	Fässler 2009: 63%
Patient expecting a therapy	Howick: 25% (NST)/17% (PP); Ferentzi: 66%
Patient explicitly requested a therapy	Howick: 43% (NST)/29% (PP); Meissner: 52% (NST)/57% (PP)
Patient requested this method	Babel 2013: 29%
To calm patient	Babel 2013: 46%; Braga-Simoes: 60%; Ferentzi: 38%; Holt: 23%; Howick: 30% (NST)/31% (PP); Kermen: 21%; Nitzan: 58%; Shah: 33%
Avoid conflict	Ferentzi: 29%; Hrobjartsson: 70%
To appease a complaining patient	Braga-Simoes: 46%; Holt: 17%; Kermen: 15%; Nitzan: 25%
Difficult patients/unwarranted complaints	Fässler 2009: 51%
Unjustified demand for a (defined) treatment	Babel 2013: 24%; Braga-Simoes: 38%; Holt: 48%; Howick: 29% (NST)/21% (PP); Kermen: 32%; Meissner: 47% (PP); Nitzan: 58%; Shah: 9%
Handling a difficult situation	Meissner: 47% (NST)/46% (PP)
Avoid discontinuing another physicians prescription	Hrobjartsson: 40%
**"Medical" reasons**	
Non-specific complaints	Babel 2013: 22%; Braga-Simoes: 47%; Fässler 2009: 64%; Ferentzi: 34%; Holt: 35; Howick: 34% (NST)/29% (PP); Kermen: 15%; Meissner: 42% (NST)/31% (PP); Nitzan: 58%; Shah: 61%
No organic background	Ferentzi: 52%
Psychological origin suspected	Ferentzi: 49%
No specific treatment available	Babel 2013: 29%, Ferentzi: 11%
All possibilities tried	Ferentzi: 35%; Holt: 33%; Kermen: 20%
Avoid telling treatment possibilities exhausted	Hrobjartsson: 36%
Option for untreatable/incurable disease	Fässler 2009: 44%; Howick: 25% (NST)/16% (PP)
To buy time/between two doses of treatment	Babel 2013: 15%; Braga-Simoes: 21%; Howick: 10% (NST)/9% (PP); Holt: 5%; Kermen: 4%; Nitzan: 8%; Shah: 6%
Avoid drug addiction	Fässler 2009: 31%; Meissner: 22% (PP)
Instead of a specific treatment avoiding harm	Babel 2013: 14%; Fässler 2009: 37%; Ferentzi: 12%
As a supplement to other therapies	Babel 2013: 54%; Braga-Simoes: 37%; Ferentzi: 30%; Howick: 27% (NST)/16% (PP); Kermen: 19%; Nitzan: 50%; Shah: 40%
As an additional treatment option	Meissner: 47% (NST)/19% (PP)
To control pain	Braga-Simoes: 15%; Howick: 13% (NST)/13% (PP); Holt: 6%; Kermen: 10%; Nitzan: 42%; Shah: 23%
As diagnostic tool	Babel 2013: 21%; Braga-Simoes: 60%; Fässler 2009: 21%; Ferentzi: 30%; Holt: 13%; Hrobjartsson: 25%; Kermen: 15%; Meissner: 25% (PP); Nitzan: 42%; Shah: 32%

NST = non-specific therapy, PP = pure placebo; percentages are usually among placebo users (however, in a minority of studies the denominator is not fully clear)
