# Supplementary material for: How often do general practitioners use placebos and non-specific interventions? Systematic review and meta-analysis of surveys
Source: PLoS One. 2018 Aug 24;13(8):e0202211. doi: 10.1371/journal.pone.0202211 (PMC6108457; doi:10.1371/journal.pone.0202211)
Supplement: S2 Table — Definitions and examples of placebo interventions given to survey participants in included studies. (PDF) [file pone.0202211.s006.pdf]

S2 Table. Definitions. Definitions and examples of placebo interventions given to survey participants in included studies

Definitions and examples of placebo interventions given to survey participants in included studies

Babel 2012: Definition (translation from Polish original by the author cited from publication) given to participants<br><br>"For our purposes, we construe [placebo interventions/nonspecific methods] to be all the medical substances, practices, and procedures whose efficacy is difficult to prove scientifically, even though they might seem efficacious. These may be pharmacological treatments of both inactive types (e.g., sugar pills and injections of saline), and active types, where the latter are used in cases in which - at least theoretically - they should have no impact on the symptoms of a patient (as with antibiotics in the treatment of viral illnesses, or vitamins taken for fatigue). Frequently, methods from natural and/or alternative medicine (e.g., homeopathy and certain physiotherapeutic procedures) are considered to be examples of [placebo interventions/ nonspecific methods]: they happen to be effective, yet the mechanism of their operation is impossible to explain scientifically.''
Babel 2013: Definition (translation from Polish original by the author cited from publication) given to participants on the questionnaire<br><br>"For our purposes, we construe nonspecific methods of treatment to be all the medical substances, practices, and procedures whose efficacy is difficult to prove scientifically, even though they might seem efficacious. These may be pharmacological treatments of both inactive types (e.g. sugar pills, injections of saline), and active types, where the latter are used in cases in which – at least theoretically – they should have no impact on the symptoms of a patient (as with antibiotics in the treatment of viral illnesses, or vitamins taken for fatigue). The same method which is specific when it is used in one case (e.g. antibiotics in the treatment of bacterial infection) may be nonspecific in other case (e.g. antibiotics in the treatment of viral illnesses). Frequently, methods from natural and/or alternative medicine (e.g. homeopathy and certain physiotherapeutic procedures) are considered to be examples of nonspecific methods of treatment: they happen to be effective, yet the mechanism of their operation is often impossible to explain scientifically."
Braga-Simoes 2017: Definition (translation from Portuguese original by the authors cited from published questionnaire) given to participants on the questionnaire [comment: while the definition provided differentiates pure and impure placebo the questions use only the general term placebo]<br><br>"Placebo: substance or treatment used for its non-specific psychological or psycho-physiological effects without specific effects for the condition being treated. The definition includes a variety of medical interventions (*e.g.* inert substances or medicines, diagnostic techniques, etc.).<br>Pure Placebo: inert substances like physiological saline that may be used for diagnostic or beneficial therapeutic effects in situations where there is no evidence of benefit for their use.<br>Impure Placebo: substances with known pharmacological activity (like Vitamin B12) that may be used in situations where they have no known evidence for effectiveness."
Fässler 2009: Definition (translation from German original by the authors cited from published questionnaire) given to participants on the questionnaire

“We define a placebo intervention as a diagnostic or therapeutic sham intervention or as an intervention with substances or physical methods which have no direct pharmacological, biochemical or physical mechanism of action according to the current standard of knowledge. The term includes a considerable variety of interventions, thus not only the administration of lactose tablets or isotonic saline solution.<br>Pure placebos are inert substances or methods such as sugar pills or isotonic saline solution. Impure placebos refer to substances or methods which have a known pharmacological or physical activity but which cannot be expected to have any direct therapeutic effects for the respective disease and in the chosen dosage, e.g. vitamin infusions for cancer or peppermint pills for pharyngitis.”
Fässler 2011: No direct definition of placebo interventions was given. Instead the author defined to modes of action (translation from German questionnaire provided by the author)<br><br>“Therapies usually have two modes of action. One mode is pharmacological (or physical) influencing the body via biochemical processes. For example, diabetic patients have to use insulin to lower their blood glucosis. The other mode of action is via non-specific (non-physical) mechanisms, also called placebo effects.”
Ferentzi 2011: Definition (as provided in the paper)<br><br>“[Placebo treatment was defined] as any kind of healing procedure that has an inherent therapeutic effect according to the patient’s view, even if there is no evidence. A distinction had been made between pure and active placebos and it has been made clear that our placebo definition includes both.”
Harris 2015: no definition given to participants
Holt 2009: unclear whether participants were given a definition
Howick 2013: Definition given to participants in the questionnaire (provided from author)<br><br>“Pure placebos are inert substances or methods such as sugar pills or isotonic saline solution.”<br>“Impure placebos are substances or methods which have a known pharmacological or physical activity for some ailments but lack proven direct specific therapeutic effects in the chosen dosage for the ailment for which they have been prescribed. These may include: Nutritional supplements for ailments unlikely to benefit from this therapy (such as vitamin C for cancer); peppermint pills for pharyngitis; antibiotics for suspected viral infections; sub-clinical doses of effective therapies Off-label uses of potentially effective therapies; Complementary and Alternative medicine (CAM) whose effectiveness is not evidence-based (including homeopathy, acupuncture); diagnostic practices, maybe on patient’s request or to calm the patient such as non-essential physical examinations”
Hrobjartsson 2003: Definition (translation from Danish original by the authors cited from published questionnaire) given to participants on the questionnaire – only physicians consenting with this definition were asked to fill in the questionnaire.<br><br>“Placebo treatment is defined in this survey as an intervention, without a specific effect on a given state, but with a possible unspecific effect.”
Kermen 2008: Definition given to participants on the questionnaire (as reported in the publication)<br><br>“A placebo was defined as ‘a substance with no known specific pharmacological activity against the condition being treated,’ the same definition recently posed by the AMA [American Medical

Association]. The placebo effect was defined as 'the therapeutic effects of a patient's expectations or beliefs.'"
Khan 2015: "Placebo is a medicine usually prescribed in place of real medicine for a variety of reasons."
Linde 2014: Examples for pure and impure placebos given to participants in the questions (as translated by authors)<br><br>[Under the heading use of typical placebos] "Have you ever used placebos (preparations without active ingredients such as placebo pills or saline injections) for treatment outside of clinical trials in your practice?"<br>[Under the heading use of non-specific treatments] "Have you ever used in your practice drugs or treatments even though you thought that these did not have any intrinsic effect against the illness of the patient or on its symptoms (e.g. antibiotics in a patient likely to suffer from a viral infection or a herbal remedy although you personally thought it is without effect on the specific symptoms)?"
Meissner 2012: Examples for pure and impure placebos given to participants in the questions (as translated by authors)<br><br>"Have you ever used medicine or therapies, even if you considered that they had no intrinsic effect (e.g. pharmacological or physical action) on the patient's disease or its symptoms?"<br>"Which forms of drugs/therapies have you already used for that purpose?"<br>"Did you ever use in your practice placebos (e.g. placebo pills, saline injections, sweeteners, etc.) outside of clinical trials?"
Nitzan 2004: no definition provided to participants
Shah 2009: unclear whether participants were given a definition

4. S-Table 1 - results of quality assessment

Results of quality assessment

Study	Item 1	Item 2	Item 3	Item 4	Item 5	Item 6
Babel 2012	no	no	unclear	no	no	yes
Babel 2013	no	no	2	no	no	yes
Braga-Simoes 2017	no	no	unclear	no	yes	yes
Fässler 2009	yes	yes	unclear	no	yes	yes
Fässler 2011	yes	yes	yes	yes	yes	no
Ferentzi 2009	yes	yes	no	no	no	yes
Harris 2011	no	no	no	no	no	no
Holt 2009	no	no	unclear	no	no	no
Howick 2013	yes	yes	unclear	yes	yes	yes
Hrobjartsson 2003	yes	yes	unclear	no	yes	no
Kermen 2010	yes	yes	unclear	yes	no	yes
Khan 2015	no	no	yes	no	no	no
Linde 2014	yes	yes	unclear	yes	yes	yes
Meissner 2012	yes	yes	unclear	yes	yes	yes
Nitzan 2004	no	no	unclear	no	no	yes
Shah 2009	no	no	unclear	no	no	no

Item 1: Was the underlying population adequately defined and relevant?
Item 2: Was the procedure to draw a sample from the population adequate?
Item 3: Is the response rate sufficiently high to rule out selection bias? (<40% = no: 40% to 70% = unclear; >70% = yes)
Item 4: Did more than 200 GPs participate?
Item 5: Was there some systematic pre-testing or validation of the questionnaire?
Item 6: Were participating GPs described?

5. S-Table 2 - numeric data on frequency of use

Proportion (95%CI) of physicians using any type of placebo, pure placebos and non-specific therapies

Study	N	Ever	Last year	≥ monthly	≥weekly
**Any Placebos**					
Babel 2012 (POL)	41	0.95 (0.82, 0.99)		0.73 (0.58, 0.84)	0.46 (0.32, 0.61)
Babel 2013 (POL)	50	0.82 (0.69, 0.90)		0.76 (0.62, 0.86)	0.50 (0.36, 0.64)
Braga-Simoes 2017 (POR)	93	0.73 (0.63, 0.81)	0.66 (0.55, 0.75)	0.34 (0.25, 0.45)	0.10 (0.05, 0.18)
Fässler 2011 (SUI)	232	0.88 (0.84, 0.92)			
Ferentzi 2009 (HUN)	169	0.83 (0.76, 0.83)	0.78 (0.71 0.84)		
Harris 2011 (CAN)	42	0.29 (0.17, 0.44)			
Holt 2009 (NZ)	157	0.71 (0.63, 0.77)	0.50 (0.42, 0.57)	0.15 (0.10, 0.22)	0.01 (0.00, 0.05)
Howick 2013 (UK)	783	0.97 (0.96, 0.98)	0.95 (0.93, 0.96)	0.89 (0.87, 0.91)	0.75 (0.72, 0.78)
Hrobjartsson 2003 (DEN)	182	0.86 (0.80, 0.91)	0.86 (0.80, 0.91)	0.48 (0.41, 0.55)	
Kermen 2010 (USA)	412	0.56 (0.51, 0.61)	0.46 (0.41, 0.51)	0.19 (0.15, 0.23)	
Khan 2015 (PK)	80	0.64 (0.53, 0.74)			
Linde 2014 (GER)	319	0.79 (0.74, 0.83)	0.76 (0.71, 0.81)	0.57 (0.52, 0.62)	0.19 (0.15, 0.24)
Meissner 2012 (GER)	208	0.88 (0.82, 0.91)	0.81 (0.75, 0.86)	0.69 (0.63, 0.75)	0.32 (0.26, 0.38)
Nitzan 2004 (ISR)	27	0.44 (0.27, 0.63)			
Shah 2009 (IND)	30	0.90 (0.73, 0.97)		0.80 (0.62, 0.91)	0.60 (0.42, 0.76)
Studies/participants		15/2555	8/2323	10/2275	8/1681
RE pooled estimate		0.79 (0.68, 0.87)	0.76 (0.61, 0.86)	0.57 (0.37, 0.74)	0.30 (0.12, 0.57)
Heterogeneity		Q=323, df=14, p<0.01, I^2=96%	Q=340, df 7, p<0.01, I^2=98%	Q=579, df=9, p<0.01, I^2=98%	Q=380, df=7, p<0.01, $I^2$99%
**Pure Placebos**					
Fässler 2009 (SUI)	166	0.18 (0.13, 0.25)		0.04 (0.02, 0.09)	0.01 (0.00, 0.05)
Howick 2013 (UK)	783	0.12 (0.10, 0.15)	0.02 (0.02, 0.04)	0.02 (0.01, 0.03)	0.01 (0.00, 0.02)
Linde 2014 (GER)	319	0.53 (0.47, 0.58)	0.46 (0.40, 0.61)	0.09 (0.07, 0.13)	0.02 (0.01, 0.05)
Meissner 2012 (GER)	208	0.49 (0.42, 0.55)	0.45 (0.39, 0.52)	0.15 (0.11, 0.21)	0.03 (0.02, 0.07)
Studies/participants		4/1476	3/1310	4/1476	4/1476
RE pooled estimate		0.30 (0.13, 0.54)	0.21 (0.03, 0.72)	0.06 (0.02, 0.15)	0.02 (0.01, 0.02)
Heterogeneity (I^2)		Q=221, df=3, p<0.01; I^2=98%	Q=201, df=2, p<0.01, I^2=99%	53.7, df=3, p<0.01, I^2=95%	Q=6.88, df=3; p=0.08, I^2=57%
**Non-specific therapies**					
Howick 2013 (UK)	783	0.97 (0.96, 0.98)	0.95 (0.93, 0.96)	0.89 (0.87, 0.91)	0.75 (0.72, 0.78)
Linde 2014 (GER)	319	0.67 (0.61, 0.72)	0.65 (0.60, 0.70)	0.53 (0.47, 0.58)	0.16 (0.13, 0.21)
Meissner 2012 (GER)	208	0.84 (0.79, 0.88)	0.75 (0.69, 0.81)	0.64 (0.58, 0.71)	0.30 (0.24, 0.37)
Studies/participants		3/1310	3/1310	3/1310	3/1310
RE pooled estimate		0.88 (0.58, 0.97)	0.83 (0.55, 0.95)	0.72 (0.44, 0.90)	0.39 (0.11, 0.76)
Heterogeneity (I^2)		Q=135, df=2, p<0.01, I^2=99%	Q=134, df=2; p<0.01, I^2=99%	Q=166, df=2, p<0.01, I^2=99%	Q0309, df=2, p<0.01, I^2=99%

n = number of studies with data

6. S-Table 3 - specific interventions used as placebos

Proportions (95%CI) of physicians having used specific interventions as placebos (part 1 – less frequently used interventions)

Study	n	Placebo Pill	NaCl	Subtherapeutic doses	Analgesics	Herbal preparations
Babel 2012 (POL)	41			0.05 (0.01, 0.18)		
Babel 2013 (POL)	50	0.01 (0.00, 0.14)	0.08 (0.03, 0.19)	0.16 (0.08, 0.29)		
Braga-Simoes 2017 (POR)	93					
Fässler 2009	166	0.05 (0.02, 0.09)	0.12 (0.08, 0.18)			
Fässler 2011 (SUI)	232					
Ferentzi 2009 (HUN)	169	0.04 (0.02, 0.08)	0.24 (0.18, 0.31)	0.12 (0.08, 0.18)	0.27 (0.21, 0.34)	
Harris 2011 (CAN)	42	0.07 (0.02, 0.20)	0.17 (0.08, 0.31)	0.12 (0.05, 0.26)	0.19 (0.10, 0.34)	0.21 (0.12, 0.36)
Holt 2009 (NZ)	157	0.02 (0.01, 0.06)	0.02 (0.01, 0.06)	0.09 (0.05, 0.26)		0.12 (0.08, 0.18)
Howick 2013 (UK)	783	0.04 (0.03, 0.06)	0.10 (0.08, 0.13)	0.46 (0.43, 0.49)		
Hrobjartsson 2003 (DEN)	182		0.05 (0.03, 0.09)			
Kermen 2010 (USA)	412	0.03 (0.02, 0.05)	0.06 (0.04, 0.09)	0.10 (0.07, 0.13)	0.09 (0.07, 0.12)	0.12 (0.09, 0.15)
Linde 2014 (GER)	319			0.08 (0.05, 0.11)	0.14 (0.10, 0.18)	0.42 (0.37, 0.48)
Meissner 2012 (GER)	208					0.61 (0.54, 0.67)
Nitzan 2004 (ISR)	27					
Shah 2009 (IND)	30					
Studies		7	8	8	4	5
RE pooled estimate		0.04 (0.03, 0.05)	0.09 (0.05, 0.15)	0.13 (0.07, 0.21)	0.16 (0.10, 0.25)	0.27 (0.12, 0.49)
Heterogeneity (I^2)		Q=5, df=6, p<0.440, I^2=0%	Q=56, df=7, p<0.01, I^2=90%	Q=290, df=7, p<0.01, I^2=94%	Q=29, df=3, p<0.01, I^2=88%	Q=180, df=4, p<0.01, I^2=98%

Proportions (95%CI) of physicians having used specific interventions as placebos (part 2 – more frequently used interventions)

Study	n	Sedatives	Supplements	Antibiotics	Homeopathic remedies	Vitamins
Babel 2012 (POL)	41		0.59 (0.43, 0.72)		0.41 (0.28, 0.57)	0.73 (0.58, 0.84)
Babel 2013 (POL)	50		0.36 (0.24, 0.50)		0.58 (0.44, 0.71)	0.60 (0.46, 0.73)
Braga-Simoes 2017 (POR)	93					
Fässler 2009	166					
Fässler 2011 (SUI)	232					
Ferentzi 2009 (HUN)	169	0.29 (0.23, 0.36)		0.17 (0.12, 0.24)		0.75 (0.68, 0.81)
Harris 2011 (CAN)	42			0.43 (0.29, 0.58)		0.48 (0.33, 0.62)
Holt 2009 (NZ)	157			0.69 (0.61, 0.76)		0.39 (0.32, 0.47)
Howick 2013 (UK)	783		0.39 (0.35, 0.42)	0.80 (0.77, 0.83)		
Hrobjartsson 2003 (DEN)	182	0.45 (0.38, 0.52)		0.70 (0.63, 0.76)		0.48 (0.41, 0.55)
Kermen 2010 (USA)	412			0.40 (0.35, 0.45)		0.23 (0.19, 0.27)
Linde 2014 (GER)	319	0.17 (0.13, 0.21)	0.35 (0.30, 0.41)	0.34 (0.29, 0.39)	0.33 (0.28, 0.39)	0.43 (0.37, 0.48)
Meissner 2012 (GER)	208	0.25 (0.20, 0.32)	0.49 (0.42, 0.55)	0.17 (0.12, 0.23)	0.52 (0.46, 0.59)	0.52 (0.45, 0.59)
Nitzan 2004 (ISR)	27					
Shah 2009 (IND)	30					
Studies		4	5	8	4	9
RE pooled estimate		0.28 (0.18, 0.41)	0.42 (0.35, 0.50)	0.45 (0.28, 0.64)	0.46 (0.34, 0.57)	0.51 (0.39, 0.62)
Heterogeneity (I^2)		Q=44, df=3, $p<0.01$, I^2=93%	Q=16, df=4, $p<0.01$, I^2=81%	Q=463, df=7, $p<0.01$, I^2=98%	Q=24, df=3, $p<0.01$, I^2=84%	Q=151, df=8, $p<0.01$, I^2=95%

7. **S-Table 4** - reasons for prescribing placebo interventions

Reasons for prescribing placebo interventions

Reason	Study: percentage agreement to reasons proposed in questionnaire
**Placebo/psycholoigcal effects**	
Eliciting placebo effects	Fässler 2009: 69%; Hrobjartsson: 48%
Possible psychological effect	Howick: 48% (NST)/51% (PP); Meissner: 79% (NST)/77% (PP)
**Group expectations and demands**	
Conform with requests of the patient	Fässler 2009: 63%
Patient expecting a therapy	Howick: 25% (NST)/17% (PP); Ferentzi: 66%
Patient explicitly requested a therapy	Howick: 43% (NST)/29% (PP); Meissner: 52% (NST)/57% (PP)
Patient requested this method	Babel 2013: 29%
To calm patient	Babel 2013: 46%; Braga-Simoes: 60%; Ferentzi: 38%; Holt: 23%; Howick: 30% (NST)/31% (PP); Kermen: 21%; Nitzan: 58%; Shah: 33%
Avoid conflict	Ferentzi: 29%; Hrobjartsson: 70%
To appease a complaining patient	Braga-Simoes: 46%; Holt: 17%; Kermen: 15%; Nitzan: 25%
Difficult patients/unwarranted complaints	Fässler 2009: 51%
Unjustified demand for a (defined) treatment	Babel 2013: 24%; Braga-Simoes: 38%; Holt: 48%; Howick: 29% (NST)/21% (PP); Kermen: 32%; Meissner: 47% (PP); Nitzan: 58%; Shah: 9%
Handling a difficult situation	Meissner: 47% (NST)/46% (PP)
Avoid discontinuing another physicians prescription	Hrobjartsson: 40%
**"Medical" reasons**	
Non-specific complaints	Babel 2013: 22%; Braga-Simoes: 47%; Fässler 2009: 64%; Ferentzi: 34%; Holt: 35; Howick: 34% (NST)/29% (PP); Kermen: 15%; Meissner: 42% (NST)/31% (PP); Nitzan: 58%; Shah: 61%
No organic background	Ferentzi: 52%
Psychological origin suspected	Ferentzi: 49%
No specific treatment available	Babel 2013: 29%, Ferentzi: 11%
All possibilities tried	Ferentzi: 35%; Holt: 33%; Kermen: 20%
Avoid telling treatment possibilities exhausted	Hrobjartsson: 36%
Option for untreatable/incurable disease	Fässler 2009: 44%; Howick: 25% (NST)/16% (PP)
To buy time/between two doses of treatment	Babel 2013: 15%; Braga-Simoes: 21%; Howick: 10% (NST)/9% (PP); Holt: 5%; Kermen: 4%; Nitzan: 8%; Shah: 6%
Avoid drug addiction	Fässler 2009: 31%; Meissner: 22% (PP)
Instead of a specific treatment avoiding harm	Babel 2013: 14%; Fässler 2009: 37%; Ferentzi: 12%
As a supplement to other therapies	Babel 2013: 54%; Braga-Simoes: 37%; Ferentzi: 30%; Howick: 27% (NST)/16% (PP); Kermen: 19%; Nitzan: 50%; Shah: 40%
As an additional treatment option	Meissner: 47% (NST)/19% (PP)
To control pain	Braga-Simoes: 15%; Howick: 13% (NST)/13% (PP); Holt: 6%; Kermen: 10%; Nitzan: 42%; Shah: 23%
As diagnostic tool	Babel 2013: 21%; Braga-Simoes: 60%; Fässler 2009: 21%; Ferentzi: 30%; Holt: 13%; Hrobjartsson: 25%; Kermen: 15%; Meissner: 25% (PP); Nitzan: 42%; Shah: 32%

NST = non-specific therapy, PP = pure placebo; percentages are usually among placebo users (however, in a minority of studies the denominator is not fully clear)
