# Supplementary material for: How often do general practitioners use placebos and non-specific interventions? Systematic review and meta-analysis of surveys
Source: PLoS One. 2018 Aug 24;13(8):e0202211. doi: 10.1371/journal.pone.0202211 (PMC6108457; doi:10.1371/journal.pone.0202211)
Supplement: S3 Table — Reasons for prescribing placebo interventions. (PDF) [file pone.0202211.s007.pdf]

S3 Table. Reasons. Reasons for prescribing placebo interventions

Reasons for prescribing placebo interventions

Reason	Study: percentage agreement to reasons proposed in questionnaire
**Placebo/psycholoigcal effects**	
Eliciting placebo effects	Fässler 2009: 69%; Hrobjartsson: 48%
Possible psychological effect	Howick: 48% (NST)/51% (PP); Meissner: 79% (NST)/77% (PP)
**Group expectations and demands**	
Conform with requests of the patient	Fässler 2009: 63%
Patient expecting a therapy	Howick: 25% (NST)/17% (PP); Ferentzi: 66%
Patient explicitly requested a therapy	Howick: 43% (NST)/29% (PP); Meissner: 52% (NST)/57% (PP)
Patient requested this method	Babel 2013: 29%
To calm patient	Babel 2013: 46%; Braga-Simoes: 60%; Ferentzi: 38%; Holt: 23%; Howick: 30% (NST)/31% (PP); Kermen: 21%; Nitzan: 58%; Shah: 33%
Avoid conflict	Ferentzi: 29%; Hrobjartsson: 70%
To appease a complaining patient	Braga-Simoes: 46%; Holt: 17%; Kermen: 15%; Nitzan: 25%
Difficult patients/unwarranted complaints	Fässler 2009: 51%
Unjustified demand for a (defined) treatment	Babel 2013: 24%; Braga-Simoes: 38%; Holt: 48%; Howick: 29% (NST)/21% (PP); Kermen: 32%; Meissner: 47% (PP); Nitzan: 58%; Shah: 9%
Handling a difficult situation	Meissner: 47% (NST)/46% (PP)
Avoid discontinuing another physicians prescription	Hrobjartsson: 40%
**"Medical" reasons**	
Non-specific complaints	Babel 2013: 22%; Braga-Simoes: 47%; Fässler 2009: 64%; Ferentzi: 34%; Holt: 35; Howick: 34% (NST)/29% (PP); Kermen: 15%; Meissner: 42% (NST)/31% (PP); Nitzan: 58%; Shah: 61%
No organic background	Ferentzi: 52%
Psychological origin suspected	Ferentzi: 49%
No specific treatment available	Babel 2013: 29%, Ferentzi: 11%
All possibilities tried	Ferentzi: 35%; Holt: 33%; Kermen: 20%
Avoid telling treatment possibilities exhausted	Hrobjartsson: 36%
Option for untreatable/incurable disease	Fässler 2009: 44%; Howick: 25% (NST)/16% (PP)
To buy time/between two doses of treatment	Babel 2013: 15%; Braga-Simoes: 21%; Howick: 10% (NST)/9% (PP); Holt: 5%; Kermen: 4%; Nitzan: 8%; Shah: 6%
Avoid drug addiction	Fässler 2009: 31%; Meissner: 22% (PP)
Instead of a specific treatment avoiding harm	Babel 2013: 14%; Fässler 2009: 37%; Ferentzi: 12%
As a supplement to other therapies	Babel 2013: 54%; Braga-Simoes: 37%; Ferentzi: 30%; Howick: 27% (NST)/16% (PP); Kermen: 19%; Nitzan: 50%; Shah: 40%
As an additional treatment option	Meissner: 47% (NST)/19% (PP)
To control pain	Braga-Simoes: 15%; Howick: 13% (NST)/13% (PP); Holt: 6%; Kermen: 10%; Nitzan: 42%; Shah: 23%
As diagnostic tool	Babel 2013: 21%; Braga-Simoes: 60%; Fässler 2009: 21%; Ferentzi: 30%; Holt: 13%; Hrobjartsson: 25%; Kermen: 15%; Meissner: 25% (PP); Nitzan: 42%; Shah: 32%

NST = non-specific therapy, PP = pure placebo; percentages are usually among placebo users (however, in a minority of studies the denominator is not fully clear
