# Supplementary material for: How often do general practitioners use placebos and non-specific interventions? Systematic review and meta-analysis of surveys
Source: PLoS One. 2018 Aug 24;13(8):e0202211. doi: 10.1371/journal.pone.0202211 (PMC6108457; doi:10.1371/journal.pone.0202211)
Supplement: S4 Table — Proportion (95%CI) of physicians using any type of placebo, pure placebos and non-specific therapies. (PDF) [file pone.0202211.s008.pdf]

S4 Table. Numeric data on frequency of use. Proportion (95%CI) of physicians using any type of placebo, pure placebos and non-specific therapies

Proportion (95%CI) of physicians using any type of placebo, pure placebos and non-specific therapies

Study	N	Ever	Last year	≥ monthly	≥weekly
**Any Placebos**					
Babel 2012 (POL)	41	0.95 (0.82, 0.99)		0.73 (0.58, 0.84)	0.46 (0.32, 0.61)
Babel 2013 (POL)	50	0.82 (0.69, 0.90)		0.76 (0.62, 0.86)	0.50 (0.36, 0.64)
Braga-Simoes 2017 (POR)	93	0.73 (0.63, 0.81)	0.66 (0.55, 0.75)	0.34 (0.25, 0.45)	0.10 (0.05, 0.18)
Fässler 2011 (SUI)	232	0.88 (0.84, 0.92)			
Ferentzi 2009 (HUN)	169	0.83 (0.76, 0.83)	0.78 (0.71 0.84)		
Harris 2011 (CAN)	42	0.29 (0.17, 0.44)			
Holt 2009 (NZ)	157	0.71 (0.63, 0.77)	0.50 (0.42, 0.57)	0.15 (0.10, 0.22)	0.01 (0.00, 0.05)
Howick 2013 (UK)	783	0.97 (0.96, 0.98)	0.95 (0.93, 0.96)	0.89 (0.87, 0.91)	0.75 (0.72, 0.78)
Hrobjartsson 2003 (DEN)	182	0.86 (0.80, 0.91)	0.86 (0.80, 0.91)	0.48 (0.41, 0.55)	
Kermen 2010 (USA)	412	0.56 (0.51, 0.61)	0.46 (0.41, 0.51)	0.19 (0.15, 0.23)	
Khan 2015 (PK)	80	0.64 (0.53, 0.74)			
Linde 2014 (GER)	319	0.79 (0.74, 0.83)	0.76 (0.71, 0.81)	0.57 (0.52, 0.62)	0.19 (0.15, 0.24)
Meissner 2012 (GER)	208	0.88 (0.82, 0.91)	0.81 (0.75, 0.86)	0.69 (0.63, 0.75)	0.32 (0.26, 0.38)
Nitzan 2004 (ISR)	27	0.44 (0.27, 0.63)			
Shah 2009 (IND)	30	0.90 (0.73, 0.97)		0.80 (0.62, 0.91)	0.60 (0.42, 0.76)
Studies/participants		15/2555	8/2323	10/2275	8/1681
RE pooled estimate		0.79 (0.68, 0.87)	0.76 (0.61, 0.86)	0.57 (0.37, 0.74)	0.30 (0.12, 0.57)
Heterogeneity		Q=323, df=14, p<0.01, I²=96%	Q=340, df 7, p<0.01, I²=98%	Q=579, df=9, p<0.01, I²=98%	Q=380, df=7, p<0.01, I²99%
**Pure Placebos**					
Fässler 2009 (SUI)	166	0.18 (0.13, 0.25)		0.04 (0.02, 0.09)	0.01 (0.00, 0.05)
Howick 2013 (UK)	783	0.12 (0.10, 0.15)	0.02 (0.02, 0.04)	0.02 (0.01, 0.03)	0.01 (0.00, 0.02)
Linde 2014 (GER)	319	0.53 (0.47, 0.58)	0.46 (0.40, 0.61)	0.09 (0.07, 0.13)	0.02 (0.01, 0.05)
Meissner 2012 (GER)	208	0.49 (0.42, 0.55)	0.45 (0.39, 0.52)	0.15 (0.11, 0.21)	0.03 (0.02, 0.07)
Studies/participants		4/1476	3/1310	4/1476	4/1476
RE pooled estimate		0.30 (0.13, 0.54)	0.21 (0.03, 0.72)	0.06 (0.02, 0.15)	0.02 (0.01, 0.02)
Heterogeneity (I²)		Q=221, df=3, p<0.01; I²=98%	Q=201, df=2, p<0.01, I²=99%	53.7, df=3, p<0.01, I²=95%	Q=6.88, df=3; p=0.08, I²=57%
**Non-specific therapies**					
Howick 2013 (UK)	783	0.97 (0.96, 0.98)	0.95 (0.93, 0.96)	0.89 (0.87, 0.91)	0.75 (0.72, 0.78)
Linde 2014 (GER)	319	0.67 (0.61, 0.72)	0.65 (0.60, 0.70)	0.53 (0.47, 0.58)	0.16 (0.13, 0.21)
Meissner 2012 (GER)	208	0.84 (0.79, 0.88)	0.75 (0.69, 0.81)	0.64 (0.58, 0.71)	0.30 (0.24, 0.37)
Studies/participants		3/1310	3/1310	3/1310	3/1310
RE pooled estimate		0.88 (0.58, 0.97)	0.83 (0.55, 0.95)	0.72 (0.44, 0.90)	0.39 (0.11, 0.76)
Heterogeneity (I²)		Q=135, df=2, p<0.01, I²=99%	Q=134, df=2; p<0.01, I²=99%	Q=166, df=2, p<0.01, I²=99%	Q0309, df=2, p<0.01, I²=99%

n = number of studies with data
