# Supplementary material for: How often do general practitioners use placebos and non-specific interventions? Systematic review and meta-analysis of surveys
Source: PLoS One. 2018 Aug 24;13(8):e0202211. doi: 10.1371/journal.pone.0202211 (PMC6108457; doi:10.1371/journal.pone.0202211)
Supplement: S5 Table — Proportions (95%CI) of physicians having used specific interventions as placebos. (PDF) [file pone.0202211.s009.pdf]

S5 Table. Specific interventions used as placebos. Proportions (95%CI) of physicians having used specific interventions as placebos.

Proportions (95%CI) of physicians having used specific interventions as placebos (part 1 – less frequently used interventions)

Study	n	Placebo Pill	NaCl	Subtherapeutic doses	Analgesics	Herbal preparations
Babel 2012 (POL)	41			0.05 (0.01, 0.18)		
Babel 2013 (POL)	50	0.01 (0.00, 0.14)	0.08 (0.03, 0.19)	0.16 (0.08, 0.29)		
Braga-Simoes 2017 (POR)	93					
Fässler 2009	166	0.05 (0.02, 0.09)	0.12 (0.08, 0.18)			
Fässler 2011 (SUI)	232					
Ferentzi 2009 (HUN)	169	0.04 (0.02, 0.08)	0.24 (0.18, 0.31)	0.12 (0.08, 0.18)	0.27 (0.21, 0.34)	
Harris 2011 (CAN)	42	0.07 (0.02, 0.20)	0.17 (0.08, 0.31)	0.12 (0.05, 0.26)	0.19 (0.10, 0.34)	0.21 (0.12, 0.36)
Holt 2009 (NZ)	157	0.02 (0.01, 0.06)	0.02 (0.01, 0.06)	0.09 (0.05, 0.26)		0.12 (0.08, 0.18)
Howick 2013 (UK)	783	0.04 (0.03, 0.06)	0.10 (0.08, 0.13)	0.46 (0.43, 0.49)		
Hrobjartsson 2003 (DEN)	182		0.05 (0.03, 0.09)			
Kermen 2010 (USA)	412	0.03 (0.02, 0.05)	0.06 (0.04, 0.09)	0.10 (0.07, 0.13)	0.09 (0.07, 0.12)	0.12 (0.09, 0.15)
Linde 2014 (GER)	319			0.08 (0.05, 0.11)	0.14 (0.10, 0.18)	0.42 (0.37, 0.48)
Meissner 2012 (GER)	208					0.61 (0.54, 0.67)
Nitzan 2004 (ISR)	27					
Shah 2009 (IND)	30					
Studies		7	8	8	4	5
RE pooled estimate		0.04 (0.03, 0.05)	0.09 (0.05, 0.15)	0.13 (0.07, 0.21)	0.16 (0.10, 0.25)	0.27 (0.12, 0.49)
Heterogeneity (I^2)		Q=5, df=6, $p<0.440$, I^2=0%	Q=56, df=7, $p<0.01$, I^2=90%	Q=290, df=7, $p<0.01$, I^2=94%	Q=29, df=3, $p<0.01$, I^2=88%	Q=180, df=4, $p<0.01$, I^2=98%

Proportions (95%CI) of physicians having used specific interventions as placebos (part 2 – more frequently used interventions)

Study	n	Sedatives	Supplements	Antibiotics	Homeopathic remedies	Vitamins
Babel 2012 (POL)	41		0.59 (0.43, 0.72)		0.41 (0.28, 0.57)	0.73 (0.58, 0.84)
Babel 2013 (POL)	50		0.36 (0.24, 0.50)		0.58 (0.44, 0.71)	0.60 (0.46, 0.73)
Braga-Simoes 2017 (POR)	93					
Fässler 2009	166					
Fässler 2011 (SUI)	232					
Ferentzi 2009 (HUN)	169	0.29 (0.23, 0.36)		0.17 (0.12, 0.24)		0.75 (0.68, 0.81)
Harris 2011 (CAN)	42			0.43 (0.29, 0.58)		0.48 (0.33, 0.62)
Holt 2009 (NZ)	157			0.69 (0.61, 0.76)		0.39 (0.32, 0.47)
Howick 2013 (UK)	783		0.39 (0.35, 0.42)	0.80 (0.77, 0.83)		
Hrobjartsson 2003 (DEN)	182	0.45 (0.38, 0.52)		0.70 (0.63, 0.76)		0.48 (0.41, 0.55)
Kermen 2010 (USA)	412			0.40 (0.35, 0.45)		0.23 (0.19, 0.27)
Linde 2014 (GER)	319	0.17 (0.13, 0.21)	0.35 (0.30, 0.41)	0.34 (0.29, 0.39)	0.33 (0.28, 0.39)	0.43 (0.37, 0.48)
Meissner 2012 (GER)	208	0.25 (0.20, 0.32)	0.49 (0.42, 0.55)	0.17 (0.12, 0.23)	0.52 (0.46, 0.59)	0.52 (0.45, 0.59)
Nitzan 2004 (ISR)	27					
Shah 2009 (IND)	30					
Studies		4	5	8	4	9
RE pooled estimate		0.28 (0.18, 0.41)	0.42 (0.35, 0.50)	0.45 (0.28, 0.64)	0.46 (0.34, 0.57)	0.51 (0.39, 0.62)
Heterogeneity (I^2)		Q=44, df=3, $p<0.01$, I^2=93%	Q=16, df=4, $p<0.01$, I^2=81%	Q=463, df=7, $p<0.01$, I^2=98%	Q=24, df=3, $p<0.01$, I^2=84%	Q=151, df=8, $p<0.01$, I^2=95%
